# Selective Stabilization of PSI-Associated Electron Transport Network Underlies Cytokinin-Mediated Delay of Leaf Senescence in Barley

**DOI:** 10.3390/ijms27167377

**Published:** 2026-08-18

**Authors:** Ernest Skowron, Magdalena Trojak, Julia Szymkiewicz

**Affiliations:** Department of Environmental Biology, Jan Kochanowski University of Kielce, Uniwersytecka 7, 25-406 Kielce, Poland; magdalena.trojak@ujk.edu.pl (M.T.); julia.szymkiewicz@ujk.edu.pl (J.S.)

**Keywords:** barley, 6-benzyladenine (BA), leaf senescence, dark-induced senescence (DIS), PSI-associated electron transport, cyclic electron flow (CEF), chloroplast proteostasis, photosynthetic protein remodeling, photosynthetic electron transport, CO_2_ assimilation

## Abstract

Leaf senescence progressively remodels the photosynthetic apparatus, leading to impaired electron transport and declining carbon assimilation. Here, we investigated how dark-induced senescence (DIS) and exogenous 6-benzyladenine (BA) affect photosystem function, cyclic electron flow (CEF), photosynthetic protein remodeling and CO_2_ assimilation in two barley (*Hordeum vulgare* L.) cultivars differing in their senescence characteristics, Carina (spring) and Lomerit (winter). DIS markedly reduced the chlorophyll content, PSI and PSII photochemistry, electron transport and CO_2_ assimilation in both cultivars, although the underlying mechanisms differed. Carina maintained higher CEF despite stronger PSII inhibition, whereas Lomerit exhibited a greater decline in CEF accompanied by stronger donor- and acceptor-side limitations of PSI. These physiological responses coincided with the selective remodeling of proteins forming the PSI-associated electron transport network, including coordinated changes in cytochrome *f*, PGRL1, NdhS, FNR and photosystem antenna proteins, indicating the functional reorganization of photosynthetic electron transport rather than uniform chloroplast protein degradation. BA delayed senescence by preserving chlorophyll, maintaining PSI and PSII activity, sustaining CEF and partially alleviating the decline in CO_2_ assimilation. The protective effects of BA were more pronounced in Carina and coincided with the more effective preservation of proteins associated with PSI-dependent electron transport. Collectively, our findings identify the selective stabilization of the PSI-associated electron transport network as a central mechanism underlying cytokinin-mediated delay of leaf senescence in barley and demonstrate that cultivar-dependent regulation of this network determines the effectiveness of cytokinin-mediated protection of photosynthesis.

## 1. Introduction

Leaf senescence is a genetically programmed developmental transition that coordinates chloroplast dismantling, nutrient remobilization and ultimately programmed cell death [1,2,3]. Because leaves constitute the primary sites of photosynthetic carbon assimilation, senescence has profound consequences for plant productivity through the progressive decline of chloroplast function and photosynthetic capacity [1]. Structural remodeling of chloroplasts accompanies this transition and includes disorganization of thylakoid membranes, loss of grana stacking, plastoglobule formation and the gradual conversion of chloroplasts into gerontoplasts [2,4]. A recent study further demonstrated that chloroplast dismantling is not a passive degenerative process but a highly coordinated program integrating loss of chloroplast proteostasis with activation of senescence-associated genes (SAGs) [5]. Accordingly, cytokinins (CKs) are recognized as major negative regulators of leaf senescence because they preserve chloroplast integrity, maintain photosynthetic competence and delay the onset of senescence-associated reprogramming [6,7].

DIS provides a robust experimental system for dissecting the physiological and molecular mechanisms underlying leaf senescence [8]. Despite its artificial nature, DIS faithfully recapitulates the principal hallmarks of developmental senescence, including chlorophyll degradation, activation of senescence-associated pathways and progressive deterioration of photosynthetic function [9,10]. In detached leaves, prolonged darkness rapidly suppresses carbon assimilation and accelerates chloroplast dismantling, resulting in coordinated remodeling of photosystems and associated electron transport components [11]. Accordingly, exogenous application of 6-benzyladenine (BA) has become a widely used approach for investigating the cytokinin-mediated regulation of senescence because it delays chlorophyll catabolism, stabilizes chlorophyll–protein complexes and preserves chloroplast function [8,12,13]. However, prolonged maintenance of chlorophyll and photosynthetic proteins has also been suggested to alter excitation energy dissipation and photosynthetic electron transport, potentially affecting susceptibility to photoinhibition when leaves are subsequently exposed to light [14].

Efficient photosynthesis depends not only on the integrity of individual thylakoid protein complexes but also on their dynamic functional interactions, which coordinate the excitation energy distribution and electron transport across the photosynthetic membrane [15,16]. Within this network, PSI occupies a central regulatory position by integrating linear and cyclic electron transport, thereby balancing ATP/NADPH production, chloroplast redox homeostasis and photoprotection [17]. The cyclic electron flow (CEF), mediated predominantly by the PGR5/PGRL1- and NDH-dependent pathways, provides the flexibility required to adjust photosynthetic electron transport to changing metabolic demands and redox conditions [17,18,19].

A recent study further indicated that regulation of CEF emerges from dynamic interactions among multiple thylakoid protein complexes rather than from the activity of independent electron transport pathways. Using co-immunoprecipitation coupled with mass spectrometry, the authors of [20] demonstrated that in Arabidopsis, PetA (cytochrome *f*, cyt*f*), the major lumenal subunit of the cytochrome *b*_6_*f* complex (cyt*b*_6_*f*), interacts with NdhS, the ferredoxin-binding subunit of the chloroplast NDH complex, via its N-terminal extension domain. Notably, this domain is highly conserved across Bryophyta, Ferns, Monocots and Dicots, suggesting that a similar regulatory interaction may also operate in cereals. The authors further identified a ~300 kDa protein complex comprising cyt*b*_6_*f* core subunits together with NdhS, ferredoxin:NADP^+^ oxidoreductase (FNR) and PGRL1, revealing a cyt*b*_6_*f*-associated regulatory module that functionally links PSI, cyclic electron transport and chloroplast redox regulation. These findings redefined CEF as part of an integrated PSI-associated electron transport network rather than a collection of independent electron transport pathways.

This emerging view of CEF as an integrated regulatory network has important implications for leaf senescence. Destabilization of the cyt*b*_6_*f* complex is expected to impair not only linear electron transport but also the coordinated regulation of PSI-dependent electron transport, thereby contributing to the progressive decline in photosynthetic performance. Consistent with this concept, senescence is accompanied by extensive remodeling of the photosynthetic apparatus, including changes in photosystem stoichiometry, selective turnover of antenna and reaction center proteins, and reorganization of excitation energy transfer [11,21]. Importantly, these processes do not occur uniformly. Instead, the sequence and extent of photosystem dismantling vary among species, genotypes and environmental conditions, indicating that PSI and PSII undergo selective rather than coordinated remodeling during senescence [22,23,24].

Low-temperature (77 K) chlorophyll fluorescence spectroscopy provides a sensitive approach for monitoring these structural changes because alterations in fluorescence emissions directly reflect changes in the organization of photosystem antenna complexes. In particular, blueshifts of PSI fluorescence have been associated with destabilization of LHCI complexes containing the Lhca proteins responsible for far-red fluorescence emission [25,26]. Likewise, the degradation or detachment of PSII-associated LHCII complexes modifies the excitation energy partitioning between PSI and PSII, thereby altering the efficiency of photosynthetic electron transport [27]. Such changes are tightly coordinated with chloroplast proteostasis and selective remodeling of photosynthetic protein complexes rather than representing indiscriminate chloroplast degradation [5]. Collectively, these observations indicate that selective remodeling of the PSI-associated electron transport network is a central feature of chloroplast reorganization during senescence. This coordinated reorganization ultimately extends beyond the light reactions to influence carbon metabolism. Progressive disruption of photosynthetic electron transport restricts both Rubisco carboxylation and ribulose-1,5-bisphosphate (RuBP) regeneration, thereby reducing photosynthetic CO_2_ assimilation [28].

Beyond delaying chlorophyll degradation, cytokinins play a central role in maintaining chloroplast integrity and photosynthetic competence during leaf senescence. They promote chlorophyll biosynthesis, stabilize chlorophyll–protein complexes, and regulate the accumulation of proteins involved in photosynthetic electron transport [29]. Consequently, cytokinin-mediated protection extends beyond retention of leaf greenness to maintenance of chloroplast proteostasis, including stabilization of thylakoid organization, photosystem integrity and chloroplast energy metabolism [7,12,19,30,31].

Our previous study demonstrated that the cytokinin-mediated delay of dark-induced senescence in barley is strongly genotype dependent and is associated with the differential preservation of photosynthetic performance, chlorophyll stability and photosynthetic protein accumulation [8]. However, the mechanistic basis of this genotype-specific response remains poorly understood. In particular, it is unknown whether differential cytokinin responsiveness reflects the selective preservation of individual photosynthetic proteins or stabilization of an integrated PSI-associated electron transport network coordinating PSI function, cyclic electron transport and chloroplast redox regulation.

Addressing this question is essential for understanding how cytokinins maintain photosynthetic competence during senescence. Barley (*Hordeum vulgare* L.) provides an excellent experimental system for this purpose owing to its agricultural importance and well-characterized senescence physiology [21,32,33]. In the present study, we investigated the effects of DIS and exogenous BA on photosystem function and organization, cyclic electron transport, photosynthetic carbon assimilation and the accumulation of proteins associated with photosynthetic electron transport in two barley cultivars differing in their senescence characteristics. We hypothesized that cultivar-dependent differences in cytokinin responsiveness are determined by the differential preservation of the PSI-associated electron transport network and that stabilization of this regulatory network underlies cytokinin-mediated maintenance of photosynthetic function during leaf senescence.

## 2. Results

### 2.1. Changes in Chlorophyll Content and SPAD During Dark-Induced Senescence

**Figure 1:**
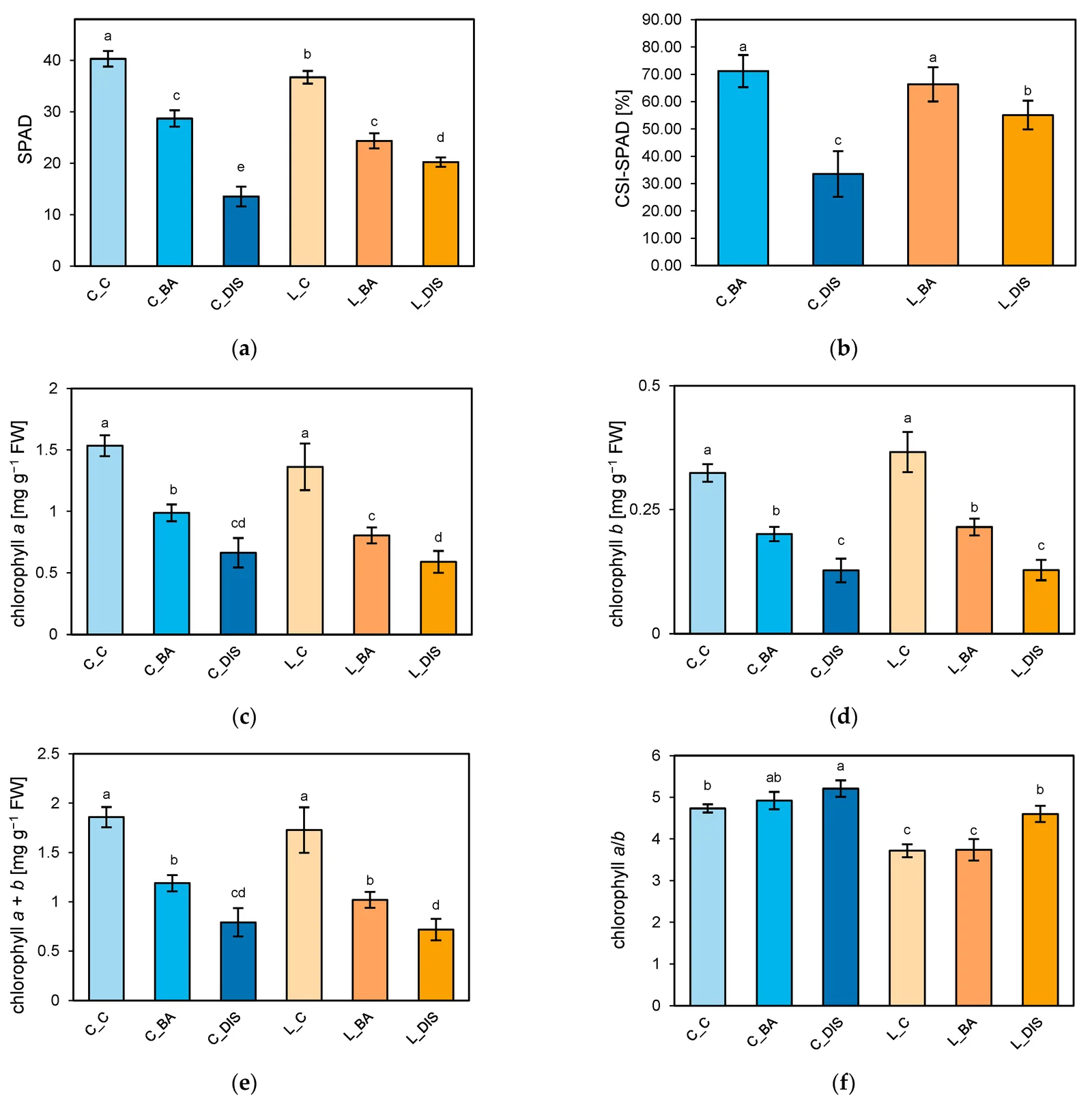
Chlorophyll content in 50 mm segments of the first true leaf of *Hordeum vulgare* L. cultivars Carina (C) and Lomerit (L) after 72 h of incubation under light in 0.2% DMSO (control, C) or in darkness in either 0.2% DMSO (DIS) or 50 μM 6-benzyladenine (BA). The chlorophyll content is expressed as the (**a**) SPAD index; (**b**) relative chlorophyll stability (CSI-SPAD), calculated as the ratio of SPAD values after 72 h of incubation to those measured before incubation; (**c**) chlorophyll *a*; (**d**) chlorophyll *b*; (**e**) total chlorophyll (Chl *a* + *b*) (all in mg g ^–1^ FW); and (**f**) chlorophyll *a*/*b* ratio. The values represent the means ± SD (*n* = 20 for (**a**,**b**); *n* = 10 for (**c**–**f**)). Different letters indicate significant differences according to Tukey’s HSD test (*p* < 0.05). FW, fresh weight.

As a result of DIS, the SPAD values decreased by 66% in Carina and 45% in Lomerit relative to the light-incubated controls (Figure 1a). BA significantly increased chlorophyll retention, with the relative chlorophyll stability (CSI-SPAD) reaching approximately 71% and 66% in Carina and Lomerit, respectively, compared with 34% and 55% in the DIS-treated leaves (Figure 1b). The total chlorophyll (Chl *a* + *b*) content declined by 57% in Carina and 58% in Lomerit following DIS (Figure 1e). BA attenuated chlorophyll loss, preserving 64% and 59% of the control’s chlorophyll content in Carina and Lomerit, respectively. Under control conditions, Carina exhibited a substantially higher Chl *a*/*b* ratio than Lomerit (4.73 vs. 3.72; Figure 1f). DIS further increased the Chl *a*/*b* ratio to 5.21 in Carina and 4.60 in Lomerit, reflecting a greater decline in Chl *b* than in Chl a (Figure 1c–f). BA largely maintained the Chl *a*/*b* ratio at control levels in both cultivars.

### 2.2. Changes in PSI/PSII Activity and Cyclic Electron Transport Induced by Senescence and BA

**Figure 2:**
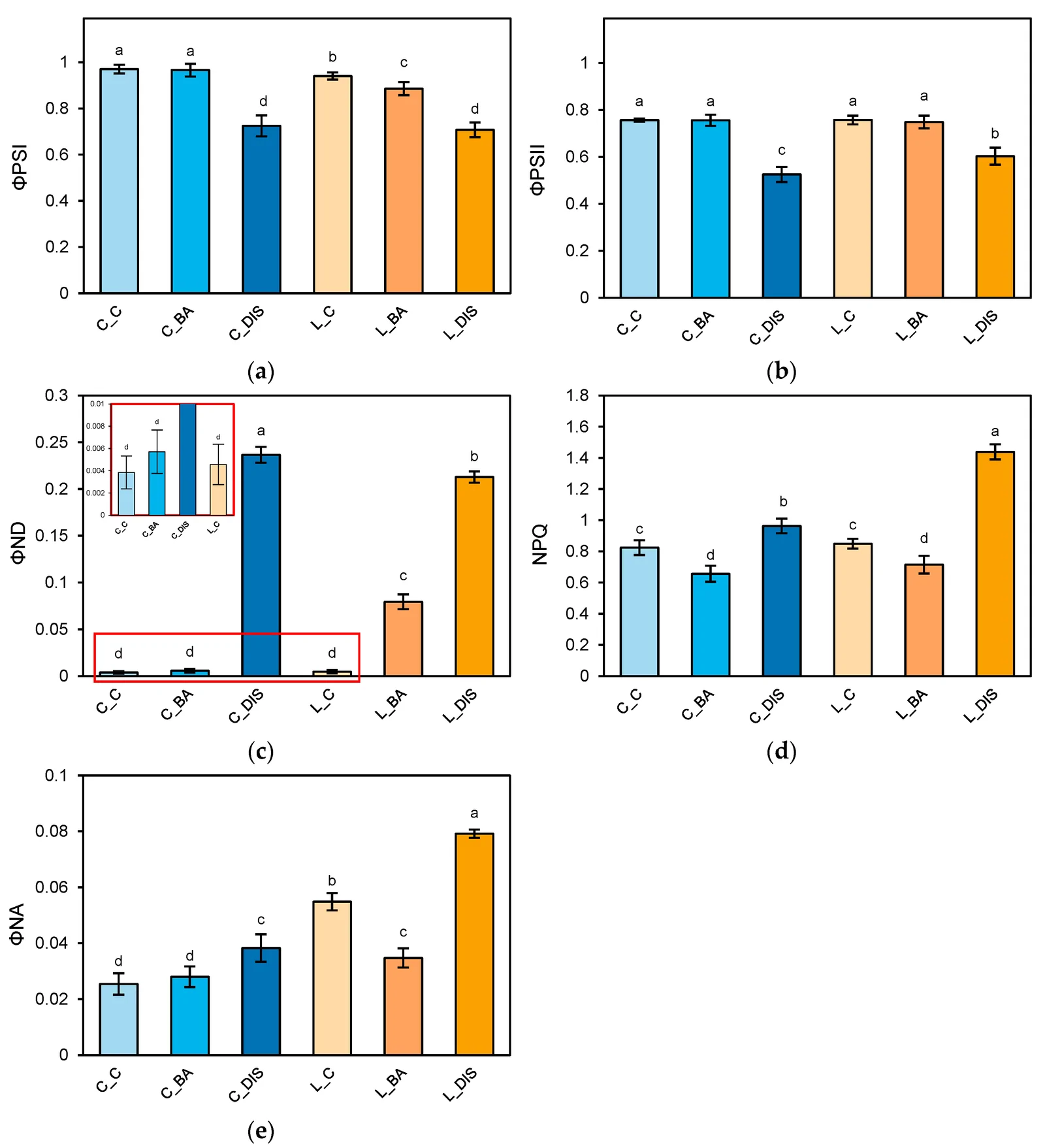
Evaluation of photosystem I (PSI; (**a**,**c**,**e**)) and photosystem II (PSII; (**b**,**d**)) activity in 50 mm segments of the first true leaf of *Hordeum vulgare* L. cultivars Carina (C) and Lomerit (L) after 72 h of incubation under light in 0.2% DMSO (control, C) or in darkness in either 0.2% DMSO (DIS) or 50 μM 6-benzyladenine (BA). (**a**) ΦPSI, effective quantum yield of PSI photochemistry; (**b**) ΦPSII, effective quantum yield of PSII photochemistry in illuminated leaves; (**c**) ΦND, quantum yield of non-photochemical energy dissipation in PSI due to donor-side limitation. Inset: Enlarged view of the low-value region of the graph; (**d**) NPQ, non-photochemical quenching; (**e**) ΦNA, quantum yield of non-photochemical energy dissipation in PSI due to acceptor-side limitation. The values represent the means ± SD (*n* = 10). Different letters indicate significant differences according to Tukey’s HSD test (*p* < 0.05).

Under the control conditions, both cultivars exhibited similar effective quantum yields of PSII (ΦPSII = 0.76), whereas ΦPSI was substantially higher, reaching 0.97 in Carina and 0.94 in Lomerit (Figure 2a,b). The high ΦPSI values observed in the control plants reflect the high physiological status of fully dark-adapted leaves grown under controlled conditions, in which PSI operates close to its maximal photochemical efficiency. Following 72 h of dark-induced senescence, ΦPSI declined by approximately 25% in both cultivars, whereas ΦPSII decreased more markedly in Carina (31%) than in Lomerit (20%) (Figure 2a,b). DIS also increased non-photochemical quenching (NPQ), with a stronger response in Lomerit than in Carina (Figure 2d). Analysis of PSI energy partitioning showed that DIS markedly increased the donor-side limitation (ΦND) in both cultivars, with the highest values observed in Carina (Figure 2c). In contrast, the acceptor-side limitation (ΦNA) remained comparatively low, but increased more strongly in Lomerit than in Carina (Figure 2e).

**Figure 3:**
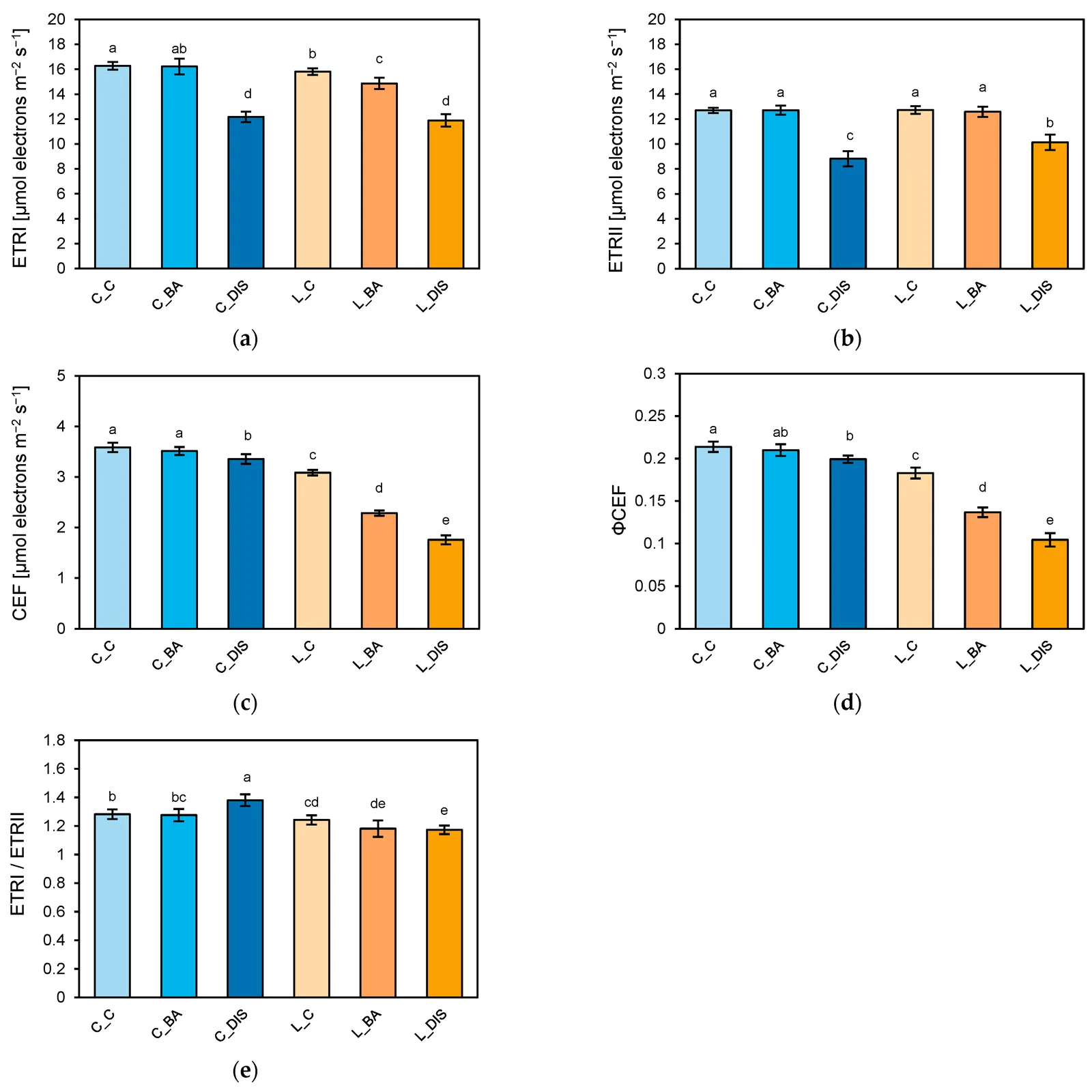
Evaluation of electron transport through photosystem I (PSI; (**a**,**c**,**d**)) and photosystem II (PSII; (**b**)), and their relative activities (**e**) in 50 mm segments of first true leaf of *Hordeum vulgare* L. cultivars Carina (C) and Lomerit (L) after 72 h of incubation under light in 0.2% DMSO (control, C) or in darkness in either 0.2% DMSO (DIS) or 50 μM 6-benzyladenine (BA). (**a**) ETRI, electron transport rate through PSI; (**b**) ETRII, electron transport rate through PSII; (**c**) CEF, cyclic electron flow around PSI; (**d**) ΦCEF, effective quantum yield of cyclic electron flow around PSI; (**e**) ETRI/ETRII, ratio of electron transport rates through PSI and PSII. Values represent means ± SD (*n* = 10). Different letters indicate significant differences according to Tukey’s HSD test (*p* < 0.05).

The decline in photosystem performance was accompanied by reduced electron transport through both PSI and PSII (Figure 3a,b). Under the control conditions, Carina exhibited a higher cyclic electron flow (CEF) and effective quantum yield of CEF (ΦCEF) than Lomerit (Figure 3c,d). During DIS, the CEF and ΦCEF declined only slightly in Carina but decreased markedly in Lomerit. Consequently, the ETRI/ETRII ratio increased in Carina but decreased in Lomerit (Figure 3e), indicating distinct cultivar-specific responses of the photosynthetic electron transport chain to senescence.

Treatment with 50 µM BA largely alleviated the senescence-induced impairment of photosynthetic performance. BA maintained ΦPSII at control levels in both cultivars and almost completely prevented a decline in ΦPSI in Carina, whereas ΦPSI in Lomerit remained only slightly below the control value (Figure 2a,b). BA also completely suppressed the DIS-induced increase in NPQ (Figure 2d), maintained ΦND and ΦNA close to the control levels in Carina, and markedly reduced both the donor- and acceptor-side limitations of PSI in Lomerit (Figure 2c,e). Similar protective effects were observed for ETRI and ETRII (Figure 3a,b). Moreover, BA largely preserved the CEF and ΦCEF in Carina, whereas only partial protection was observed in Lomerit (Figure 3c,d). Accordingly, BA largely maintained the ETRI/ETRII ratio and, overall, effectively preserved photosynthetic electron transport during DIS, with a more pronounced protective effect in Carina than in Lomerit (Figure 3e).

### 2.3. Senescence-Dependent Changes in Photosystem Organization, Relative PSI/PSII Fluorescence and Their Modulation by BA

**Figure 4:**
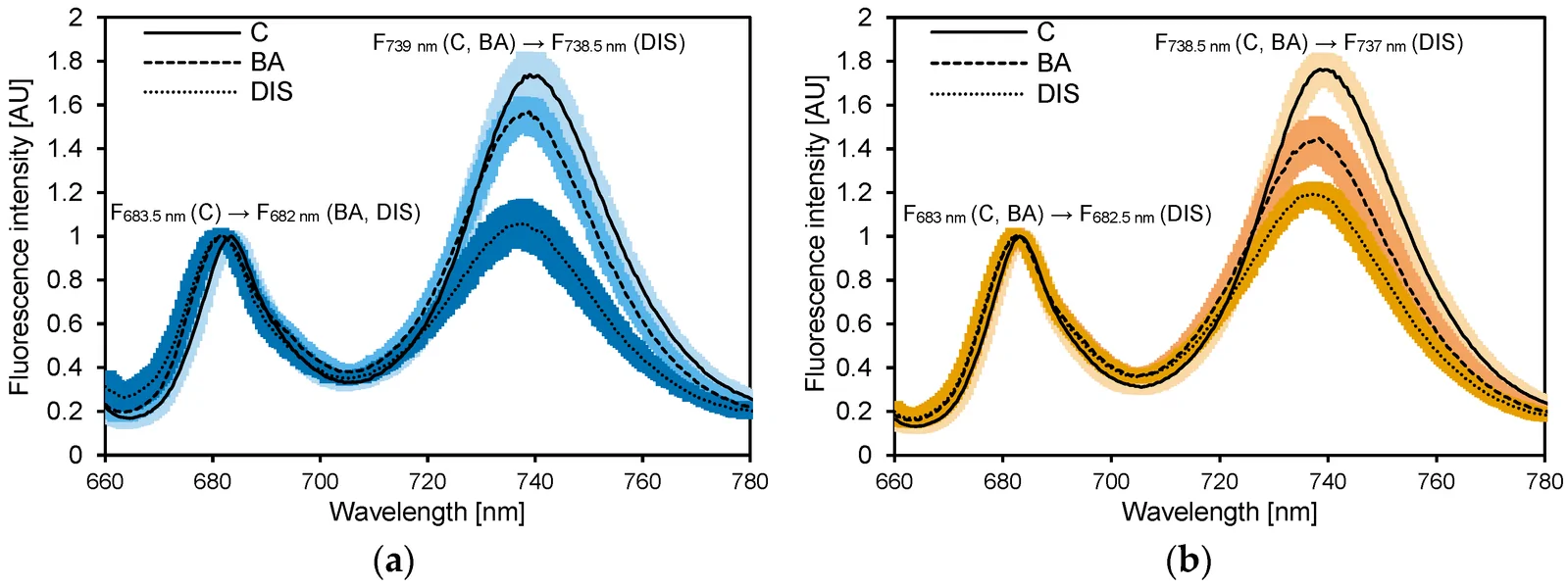
Low-temperature (77 K) chlorophyll *a* fluorescence emission spectra of leaf powder prepared from 50 mm segments of first true leaf of *Hordeum vulgare* L. cultivars (**a**) Carina and (**b**) Lomerit after 72 h of incubation under light in 0.2% DMSO (control, C) or in darkness in either 0.2% DMSO (DIS) or 50 μM 6-benzyladenine (BA). Chlorophyll fluorescence was excited at 437 nm. Spectra are normalized to maximum PSII fluorescence intensity. Curves represent mean fluorescence emission spectra (*n* = 10), with shaded areas indicating ± SD. Emission maxima corresponding to PSII and PSI are indicated together with peak shifts observed under each treatment.

The low-temperature (77 K) chlorophyll *a* fluorescence emission spectra revealed senescence-dependent remodeling of photosystem organization in both barley cultivars (Figure 4). Following 72 h of DIS, both the PSI- and PSII-associated fluorescence maxima exhibited slight blueshifts towards shorter wavelengths, indicating structural reorganization of the photosynthetic apparatus. BA largely prevented these spectral changes, maintaining control-like emission maxima in Lomerit (Figure 4b) and maintaining the PSI-associated fluorescence maximum at the control wavelength in Carina (Figure 4a).

**Figure 5:**
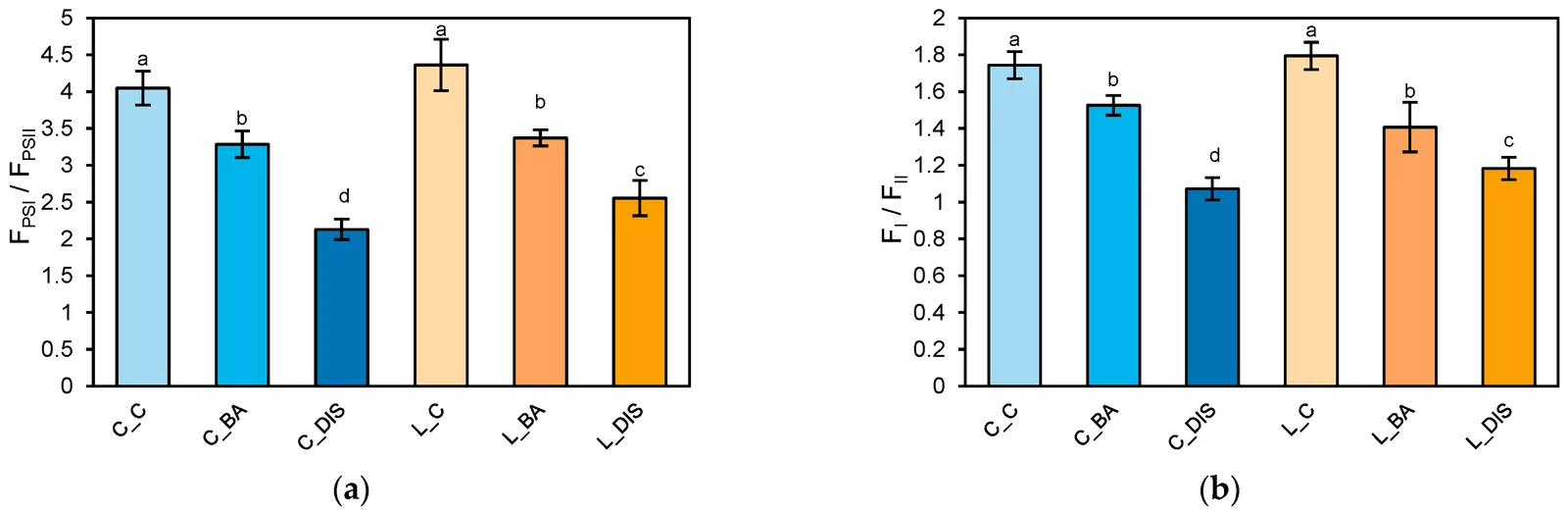
(**a**) Ratio of the integrated fluorescence emission area of PSI to PSII (F_PSI_/F_PSII_ area ratio). (**b**) Ratio of the maximum fluorescence emission of PSI to PSII (F_I_/F_II_), determined from 77 K chlorophyll *a* fluorescence emission spectra of leaf powder prepared from 50 mm segments of the first true leaf of *Hordeum vulgare* L. cultivars Carina (C) and Lomerit (L). The leaves were incubated for 72 h under light in 0.2% DMSO (control, C) or in darkness in either 50 μM BA dissolved in 0.2% DMSO (BA) or 0.2% DMSO alone (DIS). The data represent the means ± SD (*n* = 10). Different letters indicate significant differences among treatments according to Tukey’s HSD test (*p* < 0.05).

The senescence-induced changes in photosystem organization were further reflected by reductions in both the integrated PSI/PSII fluorescence area ratio (F_PSI_/F_PSII_) and the maximum fluorescence ratio (F_I_/F_II_) (Figure 5a,b). Under the control conditions, both cultivars exhibited higher PSI- than PSII-associated fluorescence, although Lomerit displayed slightly higher values for both fluorescence ratios than Carina. DIS reduced the F_PSI_/F_PSII_ ratio by approximately 48% in Carina and 41% in Lomerit (Figure 5a), while the F_I_/F_II_ ratio declined by 39% and 34%, respectively (Figure 5b). BA markedly alleviated these changes, limiting the reductions in both fluorescence ratios in the two cultivars, with a slightly stronger protective effect in Carina. Collectively, these results indicate that BA mitigated senescence-induced remodeling of photosystem organization and largely preserved the relative contribution of PSI to chlorophyll fluorescence.

### 2.4. Analysis of Net CO_2_ Assimilation and Its Modulation by Senescence and BA

**Figure 6:**
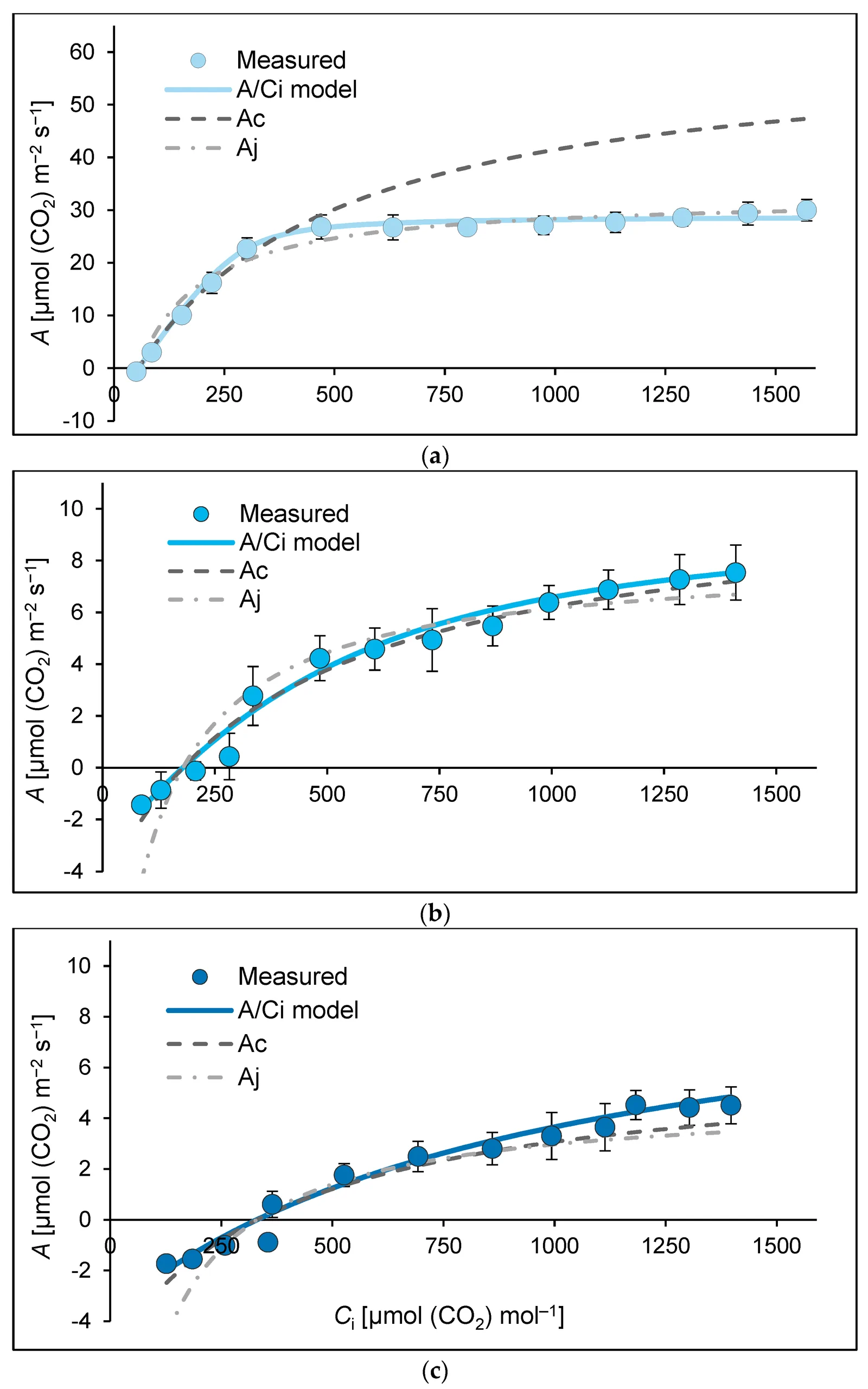
Response of net CO_2_ assimilation rate (*A*) to intercellular CO_2_ concentration (*C*_i_) in the first true leaf of *Hordeum vulgare* L. cultivar Carina after 96 h of incubation under: (**a**) light following two foliar applications of dH_2_O containing 0.2% DMSO (control, C); (**b**) darkness following two foliar applications of 50 μM 6-benzyladenine (BA) in 0.2% DMSO (BA); or (**c**) darkness following two foliar applications of dH_2_O containing 0.2% DMSO (DIS). Symbols represent means ± SD (*n* = 10). The solid-colored line represents the fitted non-rectangular hyperbola (NRH) model, whereas the dark- and light-grey dashed lines represent the Rubisco-limited (*A*_c_) and RuBP regeneration-limited (*A*_j_) regions of the fitted model, respectively.

**Figure 7:**
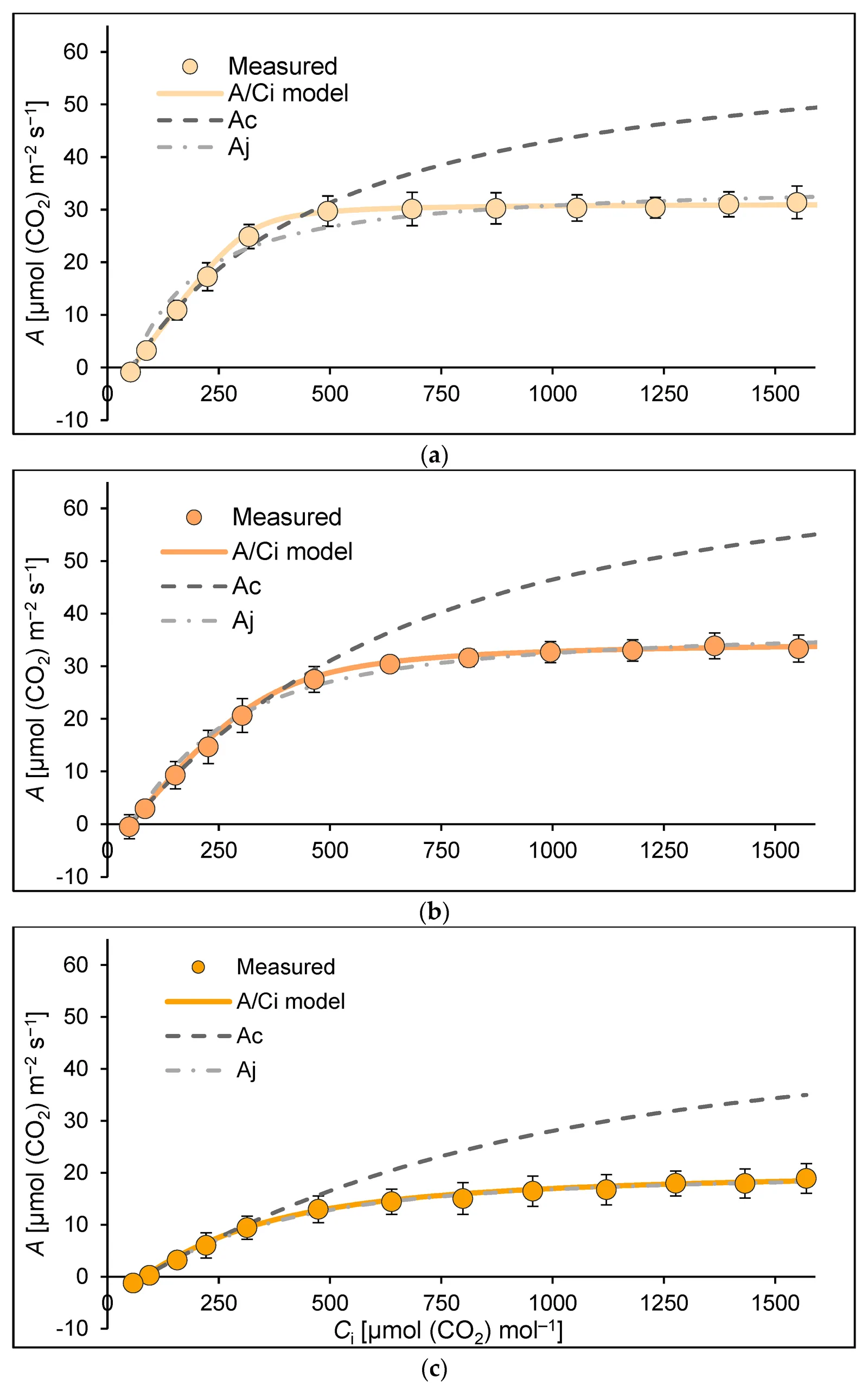
Response of net CO_2_ assimilation rate (*A*) to intercellular CO_2_ concentration (*C*_i_) in the first true leaf of *Hordeum vulgare* L. cultivar Lomerit after 96 h of incubation under: (**a**) light following two foliar applications of dH_2_O containing 0.2% DMSO (control, C); (**b**) darkness following two foliar applications of 50 μM 6-benzyladenine (BA) in 0.2% DMSO (BA); or (**c**) darkness following two foliar applications of dH_2_O containing 0.2% DMSO (DIS). Symbols represent means ± SD (*n* = 10). The solid-colored line represents the fitted non-rectangular hyperbola (NRH) model, whereas the dark- and light-grey dashed lines represent the Rubisco-limited (*A*_c_) and RuBP regeneration-limited (*A*_j_) regions of the fitted model, respectively.

CO_2_ response curve analyses were performed to identify the mechanisms underlying the decline in photosynthetic CO_2_ assimilation during DIS and its modulation by BA. Under the control conditions, the initial portion of the non-rectangular hyperbola (NRH) closely overlapped with the Rubisco-limited region (*A*_c_), whereas increasing the intercellular CO_2_ concentration (*C*_i_) progressively shifted photosynthesis towards RuBP regeneration limitation (*A*_j_) (Figure 6a and Figure 7a). Accordingly, photosynthesis was primarily Rubisco-limited at a low *C*_i_ and became increasingly limited by RuBP regeneration at a higher *C*_i_. Among the two cultivars, Lomerit exhibited a significantly higher *A*_c_ = *A*_j_ transition point than Carina (307.17 ± 12.32 versus 271.93 ± 11.79 μmol(CO_2_) mol^−1^; Table 1).

DIS markedly altered the photosynthetic limitations in a cultivar-dependent manner. In Lomerit, the *A*_c_ = *A*_j_ transition point decreased by approximately 74%, accompanied by a 40% reduction in *J*_max_ but only a 17% decline in *V*_cmax_ (Table 1). Consequently, the *J*_max_/*V*_cmax_ ratio decreased significantly, indicating that impaired RuBP regeneration became the dominant limitation to CO_2_ assimilation. In contrast, Carina exhibited a pronounced decline in both *V*_cmax_ (83%) and *J*_max_ (82%), whereas the *J*_max_/*V*_cmax_ ratio remained unchanged, indicating a simultaneous impairment of Rubisco carboxylation capacity and RuBP regeneration. As a result, the *A*_c_ = *A*_j_ transition point shifted markedly towards higher *C*_i_ values (Table 1).

The BA treatment partially alleviated the senescence-induced decline in photosynthetic capacity. In Carina, BA partially preserved *J*_max_ relative to the DIS-treated leaves; however, both *V*_cmax_ and *J*_max_ remained significantly lower than in the control (Table 1). In contrast, BA exerted a substantially stronger protective effect in Lomerit, where both *V*_cmax_ and *J*_max_ were maintained at, or slightly above, the corresponding control values. Consistent with these responses, BA markedly shifted the *A*_c_ = *A*_j_ transition point towards higher *C*_i_ values in Carina, whereas the transition point remained comparable to the control in Lomerit (Table 1).

Comparison of the fitted NRH, *A*_c_ and *A*_j_ curves further illustrated the distinct photosynthetic limitations induced by DIS in the two cultivars (Figure 6 and Figure 7). Under control conditions, the fitted NRH model exhibited a broad transition region between Rubisco-limited and RuBP regeneration-limited photosynthesis. This intermediate region disappeared following DIS in both cultivars. In Lomerit, the fitted NRH approached the *A*_j_-limited region at substantially lower *C*_i_ values than in the control, indicating an earlier onset of RuBP regeneration limitation. In contrast, Carina exhibited a pronounced increase in the chloroplastic CO_2_ compensation point (*Γ*), which increased nearly six-fold relative to the control (Table 1). Although BA substantially attenuated this increase, *Γ* remained significantly higher than in the control leaves.

### 2.5. Senescence-Dependent Remodeling of Leaf Proteins and Its Modulation by BA

#### 2.5.1. Changes in Total Soluble Protein, Carbon Assimilation Proteins and Senescence Progression During DIS

**Figure 8:**
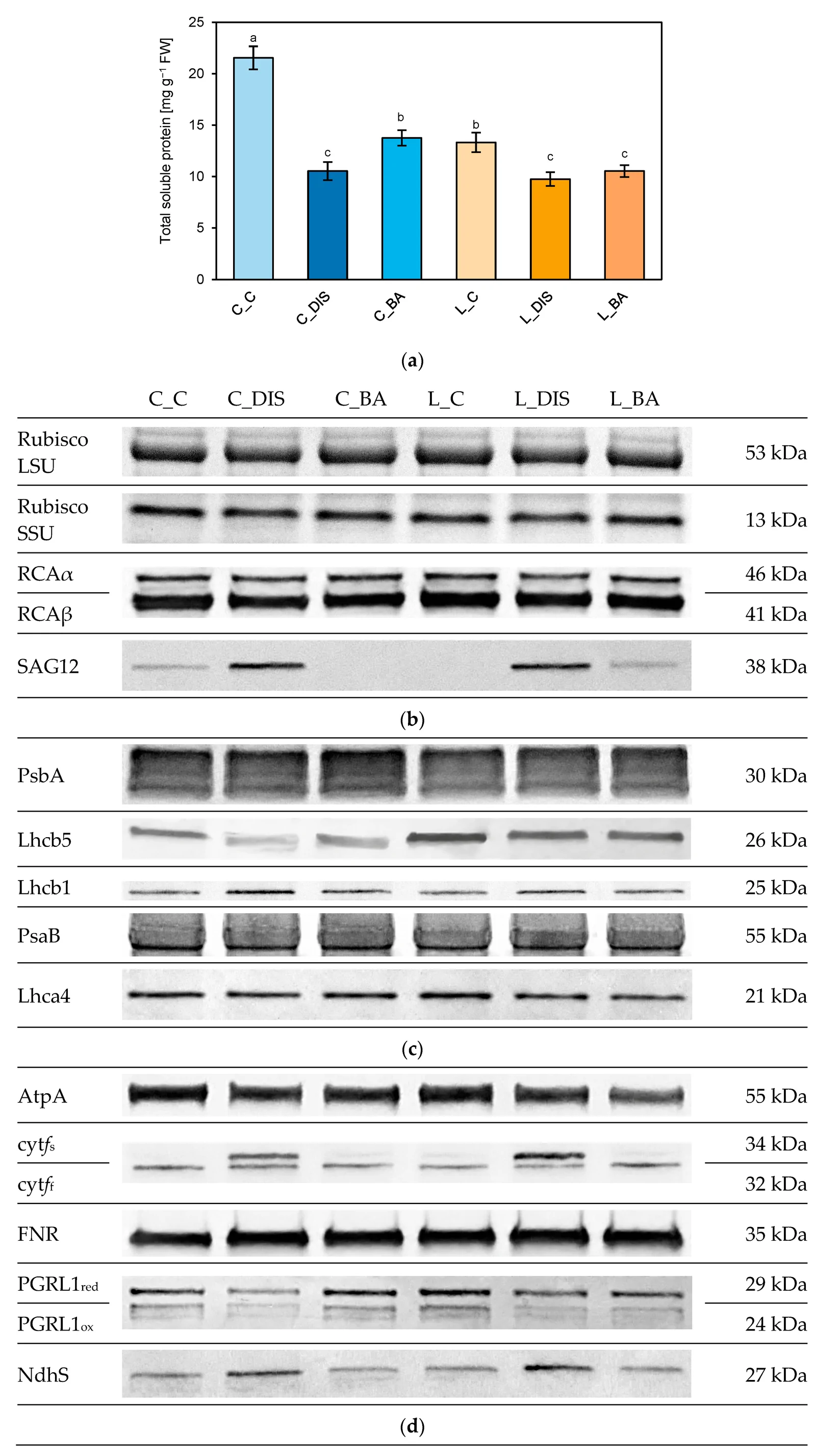

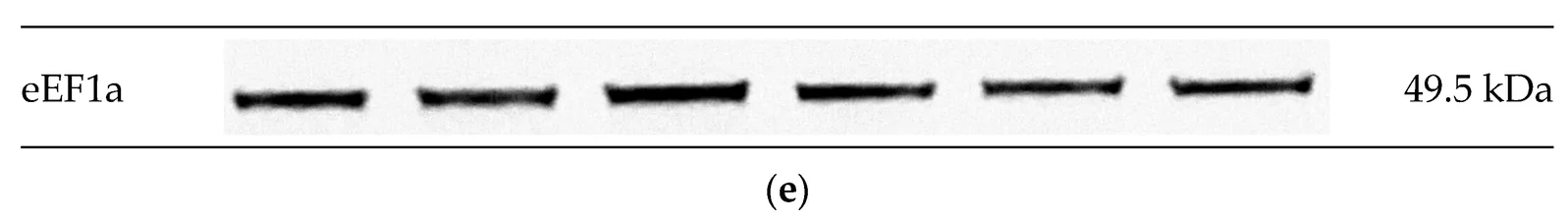
Senescence-dependent changes in the total soluble protein content and abundance of selected chloroplast proteins in the first true leaf of *Hordeum vulgare* L. cultivars Carina (C) and Lomerit (L) after 72 h of incubation under light in 0.2% DMSO (control, C), in darkness in 0.2% DMSO (DIS), or in darkness in 50 µM BA dissolved in 0.2% DMSO (BA). (**a**) The total soluble protein content is expressed as mg g^−1^ FW. (**b**) Coomassie Brilliant Blue (CBB) staining (Rubisco LSU and SSU) and Western blot analysis of proteins associated with carbon assimilation and senescence, including Rubisco activase (RCA; α- and β-isoforms) and the senescence-associated protease SAG12. (**c**) Western blot analysis of photosystem proteins, including PsbA, Lhcb5, Lhcb1, PsaB, and Lhca4. (**d**) Western blot analysis of proteins involved in photosynthetic electron transport, including the ATP synthase α-subunit (AtpA); the slow- (s) and fast (f)-migrating forms of cytochrome *f*; and the reduced (red) and oxidized (ox) forms of PGRL1, FNR, and NdhS. (**e**) For RCA, the combined signal of the α- and β-isoforms is quantified as total RCA abundance (RCA_total_). Equal amounts of total soluble protein were separated on 4–20% TGX gradient polyacrylamide gels, transferred to nitrocellulose membranes, and immunodetected. Protein loading was normalized prior to electrophoresis, and eEF1α served as the loading control. The data represent the means ± SD (*n* = 10 for (**a**); *n* = 3 for (**b**–**e**)). Different letters indicate significant differences among the treatments according to Tukey’s HSD test (*p* < 0.05). FW, fresh weight.

DIS significantly reduced the total soluble protein content in both barley cultivars (Figure 8a). Relative to the respective controls, the total soluble protein decreased by approximately 51% in Carina and 27% in Lomerit. BA significantly alleviated protein loss in Carina, maintaining the total soluble protein at approximately 64% of the control level, whereas no significant protective effect was observed in Lomerit, in which the protein content remained comparable to the DIS-treated leaves (Figure 8a).

To determine whether senescence preferentially affected specific components of the photosynthetic machinery, the relative abundance of proteins associated with carbon assimilation and senescence-related nitrogen remobilization was analyzed using equal amounts of total soluble protein (Figure 8b; Table 2). The abundance of Rubisco large (LSU) and small (SSU) subunits remained largely unchanged during senescence in both cultivars. In contrast, the total abundance of Rubisco activase (RCA_total_, RCAα + RCAβ) declined significantly, with a more pronounced reduction in Lomerit than in Carina.

The senescence-associated cysteine protease SAG12 accumulated significantly following DIS in both cultivars, confirming activation of the senescence program. Interestingly, a weak SAG12 signal was already detectable in control leaves of Carina, whereas it was absent in control leaves of Lomerit (Figure 8b; Table 2).

BA differentially affected these proteins. In Carina, BA restored the total RCA abundance to the control level, whereas only partial recovery was observed in Lomerit. Consistent with its senescence-delaying effect, BA markedly suppressed SAG12 accumulation in both cultivars, although this response was considerably stronger in Carina, where SAG12 was almost completely eliminated. In contrast, residual SAG12 abundance remained detectable in the BA-treated Lomerit leaves, indicating cultivar-dependent differences in the cytokinin-mediated regulation of senescence-associated proteolysis (Figure 8a,b; Table 2).

#### 2.5.2. Changes in Photosystem and Electron Transport Proteins

DIS also induced extensive remodeling of proteins associated with PSI and PSII organization (Figure 8c; Table 2) and photosynthetic electron transport proteins (Figure 8d; Table 2) in both barley cultivars. The abundance of the PSI antenna protein Lhca4 decreased in both cultivars, particularly in Lomerit, whereas the PSI reaction center protein PsaB remained largely unchanged throughout senescence, exhibiting only minor cultivar-dependent variations. These protein-level changes closely paralleled the previously observed reductions in PSI activity and cyclic electron flow, particularly in Lomerit. In the case of PSII, the antenna protein Lhcb5 decreased markedly in both cultivars during DIS, particularly in Carina, whereas PsbA remained remarkably stable throughout senescence. Conversely, Lhcb1 accumulated during DIS, especially in Carina, indicating differential remodeling of the PSII antenna system.

DIS profoundly remodeled the abundance of proteins associated with photosynthetic electron transport, with the most pronounced changes occurring in the components involved in PSI-dependent electron transport and cyclic electron flow (Figure 8d; Table 2). Both the reduced and oxidized forms of PGRL1 declined markedly during senescence, indicating substantial destabilization of the PGR5/PGRL1-dependent CEF pathway. Senescence was further characterized by a striking accumulation of the slower-migrating cytochrome *f* isoform (cyt*f*_s_), accompanied by a more moderate increase in the faster-migrating form (cyt*f*_f_), consistent with structural remodeling of the cyt*b*_6_*f* complex. Simultaneously, the abundance of FNR and the NAD(P)H dehydrogenase-like complex subunit S (NdhS) increased significantly, whereas the ATP synthase α-subunit (AtpA) declined in both cultivars. Collectively, these coordinated changes reveal extensive reorganization of the thylakoid electron transport machinery during dark-induced senescence.

BA differentially mitigated the senescence-associated changes in photosynthetic protein abundance, with considerably stronger effects in Carina than in Lomerit. In Carina, BA almost completely restored the abundance of Lhca4 and both PGRL1 forms to control levels, whereas PsaB abundance remained largely unchanged throughout the treatments. In contrast, BA failed to restore Lhca4 abundance and only partially restored both PGRL1 forms in Lomerit. Among the PSII-associated proteins, BA partially alleviated the loss of Lhcb5 in Carina but exerted only a limited restorative effect in Lomerit, while PsbA abundance remained essentially unchanged in both cultivars. The DIS-induced accumulation of Lhcb1 was partially attenuated by BA in both cultivars. BA also largely restored the cyt*f* isoform profile towards that of control leaves and markedly restricted the accumulation of NdhS. By contrast, FNR abundance remained above the control levels despite cytokinin treatment. Collectively, these findings demonstrate that BA preferentially preserved the proteins associated with PSI organization and PSI-dependent electron transport, thereby maintaining the integrity of the protein network supporting cyclic electron flow during senescence.

### 2.6. Correlation and Hierarchical Clustering Analyses of Photosynthetic Traits and Leaf Proteins

**Figure 9:**
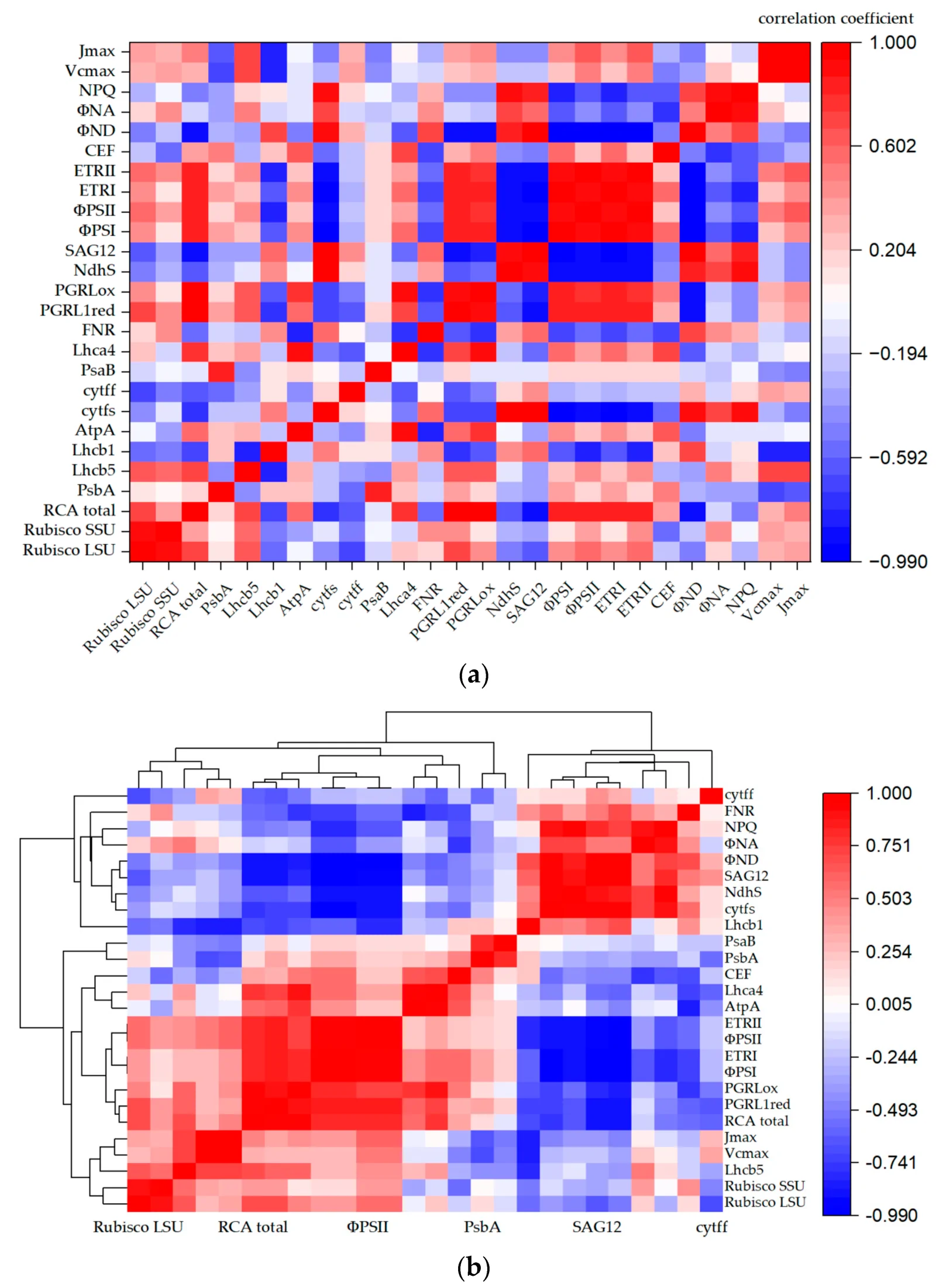
(**a**) Heatmap showing Pearson’s correlation coefficients among the photosynthetic performance parameters, leaf protein abundance, and senescence-associated markers in the first true leaf of *Hordeum vulgare* L. cultivars Carina and Lomerit after 72 h (or 96 h for gas exchange traits) of incubation under light in 0.2% DMSO (control, C), in darkness in 0.2% DMSO, or in darkness in 50 µM BA dissolved in 0.2% DMSO. The correlation coefficients (*r*) range from −1 to +1 and are represented by the color scale, where blue indicates negative correlations, white indicates no correlation, and red indicates positive correlations. (**b**) Hierarchically clustered heatmap of the same dataset. The variables are grouped using a hierarchical cluster analysis based on the similarity of their Pearson’s correlation profiles, and the resulting dendrogram illustrates the relationships among the analyzed parameters. Pearson’s correlation coefficients are calculated using the mean values obtained from 3 to 10 biological replicates for each treatment.

Pearson’s correlation analysis identified two distinct functional modules associated with either photosynthetic performance or senescence progression (Figure 9a). The photosynthesis-associated module comprised the parameters describing photosynthetic electron transport, chloroplast protein abundance, carbon assimilation and gas exchange parameters, which were predominantly connected by strong positive correlations. As expected, nearly perfect correlations were observed between ΦPSI and ETRI (*r* = 0.99998) and between ΦPSII and ETRII (*r* = 0.99999). Among the gas exchange parameters, *V*_cmax_ and *J*_max_ were also tightly associated (*r* = 0.9769), indicating a strong coordination between the Rubisco carboxylation capacity and RuBP regeneration. Notably, Rubisco activase (RCA) exhibited very strong positive correlations with both reduced and oxidized forms of PGRL1 (*r* = 0.956 and 0.951, respectively), highlighting a close functional association between Rubisco activation and cyclic electron transport.

In contrast, the senescence-associated proteins and physiological traits formed a second, clearly separated functional module. The senescence marker SAG12 showed strong positive correlations with ΦND, cyt*f*_s_ and NdhS, while exhibiting strong negative correlations with the PSI-related parameters, particularly ΦPSI and ETRI. A similar correlation pattern was observed for cyt*f*_s_, which was positively correlated with NdhS but negatively correlated with PSI photochemical activity, indicating coordinated remodeling of the PSI-associated electron transport network during senescence.

The hierarchical clustering analysis further supported this functional organization of the dataset (Figure 9b). The photosynthetic proteins and physiological parameters, including Rubisco subunits, RCA, PGRL1, PSI electron transport traits and gas exchange parameters, were clustered together and were characterized predominantly by positive intercorrelations. Notably, RCA and both Rubisco subunits were grouped together with PGRL1 and the PSI photochemical parameters, whereas SAG12, ΦND, NdhS and the cyt*f*_s_ formed a distinct senescence-associated cluster exhibiting predominantly inverse relationships with the photosynthesis-associated module. Importantly, despite the overall decline in the total soluble protein during DIS, the clustering pattern clearly separated the proteins supporting photosynthetic performance from the senescence-associated proteins, indicating selective remodeling of the chloroplast proteome rather than uniform protein degradation. Collectively, these results demonstrate a clear functional separation between a coordinated photosynthetic network supporting PSI function, cyclic electron transport and carbon assimilation, and a senescence-associated network associated with chloroplast proteome remodeling during leaf senescence.

## 3. Discussion

### 3.1. Photosystem Photochemistry and Cyclic Electron Flow During Cytokinin-Delayed Senescence

The comparable impairment of PSI photochemistry in both barley cultivars contrasts with their markedly different capacities to maintain CEF, indicating that preservation of PSI function during senescence depends primarily on sustained cyclic electron transport rather than photochemical efficiency per se. Carina maintained a substantially higher CEF than Lomerit despite stronger inhibition of PSII, indicating that sustained alternative electron transport contributed to preservation of PSI function under conditions of restricted linear electron flow. This interpretation agrees with the established role of CEF in maintaining the PSI redox balance and ATP production when linear electron transport becomes restricted [17,18]. Recent evidence further positions CEF as a dynamic regulatory network integrating the PGR5/PGRL1- and NDH-dependent pathways to coordinate ATP production with PSI photoprotection under changing physiological conditions [19].

The preferential loss of chlorophyll *b*, reflected by the increased chlorophyll *a*/*b* ratio, identifies the early dismantling of light-harvesting complexes as one of the first structural events accompanying senescence before the extensive degradation of photosynthetic reaction centers [10]. Preservation of total chlorophyll content, confirmed by both the pigment analyses and SPAD measurements, together with the partial maintenance of the chlorophyll *a*/*b* ratio, demonstrates that BA primarily preserved antenna organization rather than simply delaying chlorophyll catabolism. This interpretation is consistent with the established role of cytokinins in maintaining chloroplast integrity during senescence [7], and with the current concepts describing chlorophyll degradation and the selective remodeling of chloroplast protein complexes as tightly coordinated processes during leaf senescence [5].

The molecular progression of senescence was confirmed by the accumulation of the senescence-associated cysteine protease SAG12, linking deterioration of photosynthetic function with activation of the proteolytic machinery responsible for nutrient remobilization. The weak SAG12 signal detected in the control leaves of Carina, but not Lomerit, indicates the greater basal sensitivity of the spring cultivar to senescence induction following leaf detachment. This interpretation agrees with a previous study [34], which demonstrated that leaf excision markedly accelerates SAG12 induction during subsequent DIS, whereas intact barley leaves exhibit only minimal SAG12 accumulation after short-term darkness, with substantial induction occurring only after prolonged treatment. Conversely, the absence of detectable SAG12 in the control leaves of Lomerit is consistent with its slower senescence phenotype [8]. Although BA almost completely suppressed SAG12 accumulation in Carina, residual SAG12 remained detectable in Lomerit, indicating cultivar-dependent differences in cytokinin responsiveness. Importantly, the strong positive correlation between SAG12 abundance and ΦND identifies impairment of PSI electron transport as an integral component of senescence progression rather than a secondary consequence of prolonged darkness. Together, these observations reinforce the current concept that chloroplast dismantling, chlorophyll degradation, photosynthetic dysfunction and activation of senescence-associated genes constitute tightly coordinated processes during leaf senescence [5], supporting the view that senescence involves selective functional remodeling rather than the indiscriminate degradation of the photosynthetic apparatus.

This coordinated remodeling became particularly evident at the level of PSI electron transport. The increase in ΦND indicates a progressive restriction of electron supply to PSI [19]. In Carina, the elevated ΦND coincided with a sustained CEF and an increased ETRI/ETRII ratio, indicating preferential restriction of linear electron transport while preserving PSI function. The more pronounced increase in ΦND most likely reflects a greater donor-side limitation resulting from the stronger restriction of electron delivery from PSII, rather than the more severe impairment of PSI itself. Under these conditions, the sustained CEF likely compensated for the reduced linear electron transport by maintaining P700 in a more oxidized state, thereby supporting ATP production and preventing PSI over-reduction when linear electron transport became limiting [35]. Notably, the increase in ΦND was not accompanied by an enhanced NPQ, suggesting that the additional proton motive force generated through CEF was utilized primarily for ATP synthesis rather than thermal energy dissipation, although confirmation of this mechanism requires direct measurements of proton conductivity and ATP synthase activity [19,36]. Recent evidence further demonstrates that both the PGR5/PGRL1- and NDH-dependent pathways contribute to maintaining PSI oxidation by balancing electron partitioning and ATP/NADPH production under conditions of restricted linear electron transport [37]. However, the PGR5/PGRL1 pathway represents the predominant route of CEF under most physiological conditions, whereas the contribution of the NDH pathway becomes more pronounced mainly during prolonged stress or under low irradiance [19].

Unlike Carina, Lomerit developed both donor- and acceptor-side limitations of PSI, accompanied by a pronounced decline in CEF. The concomitant increase in NPQ indicates greater reliance on thermal energy dissipation when cyclic electron transport is no longer sufficient to maintain electron flux through PSI. Such responses are consistent with thylakoid membrane reorganization and enhanced energy dissipation mediated by remodeling of antenna complexes during stress and senescence [27]. Particularly noteworthy is the coordinated decline of both PGRL1 forms together with the accumulation of NdhS. Recent studies have demonstrated that NdhS is not merely a structural subunit of the chloroplast NDH complex but also interacts directly with the cyt*b*_6_*f* complex, forming a ferredoxin-docking module that facilitates electron transfer to the plastoquinone pool [20]. Accordingly, the increased abundance of NdhS most likely reflects compensatory remodeling of the PSI-associated electron transport network rather than activation of NDH-dependent CEF itself.

The central role of this regulatory network became particularly evident following cytokinin treatment. BA effectively restored PSI photochemistry and electron transport in both cultivars while suppressing increases in ΦND, ΦNA and NPQ. However, the extent of recovery remained strongly genotype dependent. In Carina, almost complete restoration of CEF closely paralleled recovery of PGRL1 abundance (Section 3.3), indicating preservation of the regulatory machinery controlling cyclic electron transport. In contrast, only partial restoration of PGRL1 in Lomerit was accompanied by incomplete recovery of CEF and PSI performance, reinforcing the predominant role of the PGR5/PGRL1 pathway in sustaining PSI function during cytokinin-delayed senescence. Importantly, preservation of PSI-associated electron transport did not fully restore photosynthetic carbon assimilation. In Carina, the pronounced decline in *V*_cmax_ despite the nearly complete recovery of CEF demonstrates that maintenance of cyclic electron transport alone is insufficient to sustain photosynthetic performance. The coordinated preservation of Calvin-cycle metabolism is also required.

### 3.2. Photosystem Remodeling During Leaf Senescence Revealed by Low-Temperature Chlorophyll Fluorescence

The 77 K chlorophyll fluorescence spectra demonstrate that DIS preferentially remodels photosystem organization rather than uniformly destabilizing the photosynthetic apparatus. Although both cultivars exhibited shifts in PSI- and PSII-associated fluorescence, the pattern of structural reorganization differed substantially. Carina displayed more pronounced changes in PSII-associated fluorescence, exhibiting a 1.5 nm blueshift of the PSII fluorescence maximum in both the DIS- and BA-treated leaves. In contrast, remodeling in Lomerit predominantly affected PSI, with DIS inducing a 1.5 nm blueshift of the PSI fluorescence maximum. Similar PSI blueshifts have previously been linked to destabilization of LHCI antenna complexes containing Lhca4 and red chlorophyll forms [25,26]. Likewise, studies using barley mutants deficient in LHCI proteins have demonstrated that reduced Lhca4 accumulation results in pronounced blueshifts accompanied by diminished PSI fluorescence emission [38,39].

Recent structural studies have further established that the stable coupling of LHCI to the PSI core is essential for efficient excitation energy transfer and the long-term stability of PSI. Disruption of LHCI interactions preferentially compromises antenna function without destabilizing the PSI reaction center itself [40]. Consistent with these structural observations, our immunoblot analyses revealed a preferential reduction inLhca4 abundance, particularly in Lomerit, while the PSI core protein PsaB remained comparatively stable during DIS. Together, the fluorescence and immunoblot data demonstrate that senescence preferentially targets PSI-associated antenna proteins while largely preserving the structural integrity of the PSI reaction center.

The decline in the F_I_/F_II_ ratio further supports the preferential remodeling of PSI-associated structures during senescence. In Carina, the greater reduction in F_I_/F_II_ primarily reflected the stronger functional restriction of PSII rather than destabilization of the PSI reaction center. By contrast, Lomerit combined a greater destabilization of PSI antenna proteins with reduced CEF and impaired PSI photochemistry, identifying the structural remodeling of the PSI antenna as the principal limitation to PSI function during senescence. The slightly different magnitudes of change observed for the F_PSI_/F_PSII_ and F_I_/F_II_ ratios are consistent with the complementary nature of these parameters, as F_PSI_/F_PSII_ reflects the integrated fluorescence emission of both photosystems, whereas F_I_/F_II_ represents the ratio of their fluorescence maxima. Together, these complementary analyses indicate that the two cultivars underwent distinct patterns of thylakoid membrane remodeling during senescence.

Preservation of photosystem organization emerged as one of the most prominent effects of cytokinin treatment. BA largely prevented DIS-induced changes in fluorescence characteristics, with almost complete stabilization of PSI and PSII fluorescence maxima in Carina and pronounced protection of PSI organization in Lomerit. These observations reinforce the established role of cytokinins in maintaining chloroplast integrity during senescence [7,13], and further support the concept that cytokinin-mediated preservation of PSI-associated electron transport constitutes an important component of delayed senescence. Moreover, the close correspondence between preservation of Lhca4 abundance and stabilization of PSI fluorescence provides complementary evidence that cytokinin maintains chloroplast proteostasis primarily through stabilization of photosynthetic protein complexes. This conclusion directly links the structural changes revealed by the 77 K fluorescence with the selective remodeling of photosynthetic proteins discussed in the following section.

### 3.3. Selective Remodeling of Photosynthetic Proteins During Senescence and Its Modulation by BA

The structural changes revealed by the 77 K fluorescence were paralleled by the highly selective remodeling of proteins associated with photosystem organization and photosynthetic electron transport. Rather than promoting uniform degradation of chloroplast proteins, senescence preferentially affected the regulatory and antenna components while largely preserving the reaction center proteins. The most pronounced changes involved Lhca4 and both forms of PGRL1, whereas the PSI core protein PsaB remained comparatively stable throughout DIS. Similar selective remodeling was evident within PSII, where Lhcb5 declined while Lhcb1 accumulated, demonstrating a differential reorganization of the antenna system rather than coordinated degradation of LHCII proteins. In parallel, the abundance of FNR and the NDH complex subunit S increased substantially in both cultivars, further indicating that senescence remodels the PSI-associated electron transport network instead of simply dismantling chloroplast proteins. Comparable cultivar-dependent patterns of photosystem remodeling have previously been reported during barley leaf senescence [21,41].

The contrasting behavior of proteins associated with cyclic electron transport further identified selective remodeling of the PSI-associated electron transport network as a central feature of senescence. Under the non-reducing electrophoretic conditions used in this study, two PGRL1-immunoreactive bands were resolved. Consistent with the previous study [42], these bands were interpreted as the reduced (PGRL1_red_) and oxidized (PGRL1_ox_) forms of PGRL1, whose electrophoretic mobility depends on the redox state of the conserved cysteine pair within the protein, and has been linked to regulation of ferredoxin-dependent cyclic electron transport [42]. Accordingly, the pronounced decline of both PGRL1 forms closely paralleled the reduction in CEF, particularly in Lomerit, consistent with the established role of the PGR5/PGRL1 pathways as the predominant routes of cyclic electron transport under physiological conditions [17,43]. Recent studies have further demonstrated that the PGR5/PGRL1- and NDH-dependent pathways operate cooperatively rather than independently, with PGRL1 providing rapid regulation of electron partitioning and the NDH complex contributing to long-term maintenance of PSI redox homeostasis [44]. Within this framework, the present study was designed to investigate remodeling of the PSI-associated electron transport network during senescence rather than the relative contribution of individual CEF pathways.

Accordingly, the reciprocal changes in PGRL1 and NdhS abundance observed here are more consistent with coordinated remodeling of the PSI-associated electron transport network than with compensatory regulation of two independent CEF pathways. This interpretation is reinforced by the recent demonstration that NdhS physically interacts with the cyt*b*_6_*f* complex, forming a ferredoxin-docking module that facilitates electron transfer to the plastoquinone pool and functionally links NDH with cyt*b*_6_*f* [20].

An additional observation concerns the occurrence of two cyt*f*-immunoreactive forms displaying distinct electrophoretic mobilities. Although their molecular identity remains unresolved, previous biochemical studies have demonstrated that cyt*b*_6_*f* complexes isolated from grana and stroma lamellae differ in their protein composition and are associated with distinct functional domains of the thylakoid membrane [45,46]. Within this framework, the senescence-associated increase in the slower-migrating cyt*f* form (cyt*f*_s_) is compatible with remodeling of functionally distinct cyt*b*_6_*f* populations accompanying thylakoid membrane reorganization. This interpretation agrees with the well-established decompaction of grana during dark-induced senescence [34] and with the preservation of thylakoid organization by cytokinins [7,31]. Although direct evidence is lacking, these observations raise the possibility that cytokinin limits the senescence-associated redistribution of cyt*b*_6_*f* complexes between granal and stromal thylakoid domains, thereby contributing to maintenance of the PSI-associated electron transport network.

Recent work has further positioned FNR as an integral component of this regulatory network rather than solely the terminal enzyme of linear electron transport. In addition to catalyzing NADP^+^ reduction, the membrane-associated FNR interacts with both PSI and the cyt*b*_6_*f* complex. These interactions promote ferredoxin-dependent cyclic electron transport and dynamically regulate electron partitioning between linear and cyclic pathways [47]. Consistent with this model, increased FNR abundance during DIS most likely reflects adaptive reorganization of electron distribution rather than enhanced capacity for linear electron transport. Moreover, the simultaneous decline in Lhca4 and accumulation of NdhS agrees with current structural models of the PSI–NDH supercomplex, in which the minor antenna protein Lhca5 replaces Lhca4 to facilitate NDH association with PSI [48]. The reduction in Lhca4 abundance alone is unlikely to account fully for the observed decline in PSI function. Instead, the coordinated remodeling of Lhca4 together with changes in PGRL1, cyt*f*, NdhS, FNR and CEF indicates that senescence progressively reorganizes the PSI-associated electron transport network rather than impairing individual components in isolation. Although neither Lhca5 abundance nor PSI–NDH supercomplex formation was examined directly, these observations suggest that senescence may involve partial reorganization of PSI antenna composition accompanying remodeling of the PSI-associated electron transport network.

The central role of the PSI-associated electron transport network became particularly evident following cytokinin treatment. BA largely prevented senescence-induced remodeling of this network by stabilizing Lhca4, both PGRL1 forms and the cyt*b*_6_*f*-associated protein profile, while simultaneously restricting NdhS accumulation. Preservation of the cyt*f* isoform pattern further indicates that cytokinin maintains structural organization of the cyt*b*_6_*f* complex during senescence. In Carina, the almost complete restoration of PGRL1 closely paralleled recovery of CEF and PSI photochemistry, whereas the weaker preservation of PGRL1 in Lomerit coincided with only a partial recovery of cyclic electron transport. Together, these findings identify preservation of the PGR5/PGRL1-dependent regulatory module as a central mechanism underlying cytokinin-mediated maintenance of PSI function and cyclic electron transport during senescence, rather than a mechanism sufficient to restore overall photosynthetic performance. The strong positive relationships between PGRL1 abundance, PSI activity, CEF and overall photosynthetic performance further reinforce this conclusion.

Collectively, our results support a model in which senescence does not simply suppress photosynthetic electron transport but selectively remodels a PSI-associated electron transport network comprising LHCI, PGRL1, cyt*b*_6_*f*, NdhS and FNR. Within this network, cytokinin preserves both the structural organization and regulatory connectivity, thereby sustaining PSI photochemistry and cyclic electron transport despite progressive senescence [20]. Together with our previous demonstration that BA alleviates oxidative stress and enhances antioxidant protection during dark-induced senescence [8], these findings indicate that cytokinin-mediated preservation of the photosynthetic apparatus involves coordinated stabilization of both the PSI-associated electron transport network and chloroplast redox homeostasis.

### 3.4. CO_2_ Assimilation and Biochemical Limitations of Photosynthesis During Senescence

However, maintaining the PSI-associated electron transport network alone was insufficient to preserve overall photosynthetic performance, as carbon assimilation remained constrained during senescence. Consequently, the two cultivars exhibited distinct biochemical limitations despite experiencing a similar decline in photosynthetic performance. Lomerit exhibited a predominant reduction in *J*_max_, indicating that impaired RuBP regeneration represented the major limitation to carbon assimilation, consistent with its pronounced decline in PSI activity and CEF. By contrast, Carina displayed comparable reductions in *J*_max_ and *V*_cmax_, demonstrating that senescence simultaneously constrained electron transport-dependent RuBP regeneration and the Rubisco carboxylation capacity. Notably, the more pronounced decline in RCA abundance observed in Lomerit did not preclude substantial recovery of *J*_max_ following BA treatment, indicating that Rubisco activase abundance alone was insufficient to explain the cultivar-specific differences in carbon assimilation. Together, these observations reinforce the concept that efficient photosynthesis requires tight coordination between chloroplast electron transport and Calvin-cycle metabolism rather than preservation of either process alone [49,50].

This functional uncoupling was further supported by the biochemical characteristics of Rubisco. Despite only minor changes in the abundance of Rubisco large and small subunits, DIS markedly increased the chloroplastic CO_2_ compensation point (*Γ*), particularly in Carina. An elevated *Γ* is generally associated with enhanced photorespiration and increased oxygenation activity of Rubisco under conditions restricting carbon assimilation [28,51]. Moreover, the simultaneous decline in *J*_max_, *V*_cmax_ and Rubisco activase, together with the largely unchanged abundance of Rubisco subunits, indicates that impaired Rubisco activation rather than degradation of the carboxylase became the primary biochemical limitation during senescence. This interpretation is fully consistent with recent evidence identifying Rubisco activase as an early target of dark-induced senescence, whose decline precedes the substantial degradation of Rubisco itself [52].

The differential response to BA further demonstrated that preservation of photosynthetic electron transport and recovery of carbon metabolism are only partially coupled during senescence. In Lomerit, BA restored both *J*_max_ and *V*_cmax_ to values comparable to, or slightly exceeding, those of the control, indicating coordinated preservation of electron transport and Calvin-cycle activity. By contrast, BA preferentially restored *J*_max_ in Carina, whereas *V*_cmax_ remained significantly below the control despite the complete recovery of Rubisco activase abundance. In Carina, these observations indicate that BA preferentially preserved RuBP regeneration. Notably, this functional uncoupling between recovery of PSI-associated electron transport and carbon assimilation was observed only in Carina, whereas Lomerit exhibited coordinated recovery of both *J*_max_ and *V*_cmax_. This cultivar-specific response is consistent with our previous findings, in which BA also only partially restored CO_2_ assimilation in Carina despite pronounced preservation of the photosynthetic apparatus [8]. Nevertheless, full restoration of Rubisco carboxylation capacity appears to require additional metabolic processes beyond maintenance of the photosynthetic electron transport network. The biochemical basis of this cultivar-specific limitation remains unresolved, as neither the Rubisco activation state nor post-translational regulation of Rubisco or Rubisco activase was examined in the present study. This interpretation is consistent with previous reports demonstrating that cytokinins primarily sustain photosynthesis by preserving chloroplast function and photosynthetic electron transport rather than by preventing Rubisco degradation [49]. Consistent with this model, the close association between preservation of PGRL1 abundance, maintenance of CEF, recovery of *J*_max_ and stabilization of Rubisco activase identifies the coordinated regulation of the light reactions and carbon metabolism as a prerequisite for sustaining photosynthetic function during leaf senescence [50,52].

## 4. Materials and Methods

### 4.1. Plant Material, Growth Conditions and Experimental Design

Two barley (*Hordeum vulgare* L.) cultivars differing in their senescence characteristics, Carina (a spring cultivar exhibiting rapid senescence) and Lomerit (a winter cultivar exhibiting slower senescence), were selected based on previous physiological and biochemical studies [8,21,53]. Seeds were stratified on moist filter paper in darkness at 4 °C for 2 d and subsequently germinated for 2 d at 21 °C. Uniform seedlings were transplanted into trays containing peat-based substrate (pH 5.5–6.0) supplemented with perlite, silica sand, and slow-release fertilizer (Scotts Poland, Warsaw, Poland). Plants were cultivated under controlled-environment conditions with 16 h photoperiod, day/night temperatures of 23 ± 1 °C and 20 ± 1 °C, relative humidity of 50–60%, and an ambient CO_2_ concentration (435 ± 20 μmol mol^−1^). Illumination was provided by PX256 PxCrop LED fixtures (PXM, Podłęże, Poland), providing photosynthetic photon flux density (PPFD) of 150 μmol m^−2^ s^−1^. Emission spectrum consisted of red (671 nm), green (524 nm), and blue (438 nm) wavelengths in 9:9:8 ratio, measured using GL SPECTIS 5.0 Touch spectroradiometer (GL Optic, Weilheim/Teck, Germany). Plants were irrigated as required throughout cultivation. Unless otherwise stated, all measurements were performed on first fully expanded leaf of 14-day-old plants (Zadoks growth stage 13), when two leaves were fully expanded and third leaf had emerged [54].

### 4.2. Dark-Induced Senescence and Cytokinin Treatments

Leaf samples were collected between 08:00 and 10:00 AM to minimize diurnal variation in physiological status and protein abundance [55]. After removing apical (15–20 mm) and basal (15–20 mm) portions, 50 mm leaf segments were excised and floated adaxial side up on Petri dishes containing 15 mL of distilled water (dH_2_O) supplemented with 0.2% (*v*/*v*) DMSO (Honeywell International Inc., Charlotte, NC, USA). Depending on treatment, incubation medium was supplemented with 50 μM 6-benzyladenine (BA) (Sigma-Aldrich, St. Louis, MO, USA) or left untreated according to protocol described previously [8]. Detached leaf segments used for pigment, fluorescence and immunoblot analyses were incubated for 72 h either under growth light conditions (control, C) or in complete darkness to induce senescence (DIS). Petri dishes were sealed throughout incubation period and gently agitated once daily. Following treatment, samples designated for biochemical analyses were immediately frozen in liquid nitrogen and stored at −80 °C until further processing [56].

For gas exchange measurements, senescence was induced on attached leaves. First fully expanded leaves were individually enclosed in light-impermeable but air-permeable covers for 96 h while plants remained under the standard growth conditions. Prior to shading, leaves were sprayed twice at 3 h intervals (between 07:00 and 10:00 AM) with either distilled water (control and DIS treatments) or 50 μM 6-benzyladenine (BA) at 1 mL per leaf per application [57]. All foliar solutions contained 0.01% (*v*/*v*) Tween 20 to improve leaf wetting and solution uptake. This attached-leaf darkening system was used exclusively for *A*/*C*_i_ response curve measurements, whereas all other physiological, biochemical and molecular analyses were performed using detached leaf segments. Both detached- and attached-leaf darkening systems are well-established experimental approaches for investigating dark-induced leaf senescence [58,59].

### 4.3. Determination of Photosynthetic Pigments and Relative Chlorophyll Content

Photosynthetic pigments were quantified spectrophotometrically after extraction with DMSO. Frozen leaf samples were homogenized in liquid nitrogen, and pigments were extracted with DMSO (1.5 mL per 10 mg fresh weight) at 65 °C for 3 h. Absorbance was recorded at 649 and 665 nm using microplate spectrophotometer (Mobi, MicroDigital Co., Seongnam, Republic of Korea), and chlorophyll *a* and chlorophyll *b* were calculated according to previous assays [60,61]. Ten biological replicates were analyzed for each treatment. Relative chlorophyll content was assessed non-destructively using SPAD-502 chlorophyll meter (Minolta Camera Co., Osaka, Japan). SPAD measurements were performed immediately before destructive sampling for pigment analysis. Three measurements were taken along each leaf segment and averaged to obtain single SPAD value.

### 4.4. Simultaneous Measurements of PSI and PSII Photochemistry

PSI and PSII photochemistry were simultaneously assessed using Dual-PAM-100 fluorometer (Heinz Walz GmbH, Effeltrich, Germany). PSI photochemistry was monitored from changes in P700 absorbance measured as differential signal between 830 and 875 nm, whereas PSII photochemistry was determined from chlorophyll fluorescence. Following 30 min of dark adaptation, chlorophyll fluorescence was recorded using modulated measuring light (620 nm, 3 μmol m^−2^ s^−1^). Saturating pulses (635 nm; 10,000 μmol m^−2^ s^−1^; 300 ms) and far-red light (720 nm) were applied to determine maximal fluorescence and P700 oxidation signals. Measurements were performed under red actinic light (635 nm) at intensity of 115 μmol m^−2^ s^−1^. Effective quantum yields of PSI (ΦPSI) and PSII (ΦPSII), electron transport rates through PSI (ETRI) and PSII (ETRII), non-photochemical quantum yields associated with donor- and acceptor-side limitation of PSI (ΦND and ΦNA, respectively), and non-photochemical quenching (NPQ) were calculated according to previous protocol [62]. Cyclic electron flow around PSI (CEF) was estimated as difference between ETRI and ETRII [63], whereas effective quantum yield associated with cyclic electron flow (ΦCEF) was calculated as difference between ΦPSI and ΦPSII (Table 3). For ETR calculations, leaf absorptance (Abs.) was assumed to be 0.84, as commonly applied for green leaves. This assumption was verified using Maxi IMAGING PAM M Series system (Walz, Germany), which showed absorptance values close to 0.84 across all experimental treatments. Measurements were performed on detached segments of first fully expanded leaf using ten independent biological replicates per treatment.

### 4.5. Low-Temperature (77 K) Chlorophyll Fluorescence Spectroscopy

Low-temperature (77 K) chlorophyll fluorescence spectroscopy was used to assess photosystem organization and relative PSI/PSII fluorescence emission. Frozen leaf samples were ground to fine powder in liquid nitrogen and suspended in 50 mM HEPES buffer (pH 7.5) containing 330 mM sorbitol. Suspensions were adjusted to uniform chlorophyll concentration to minimize fluorescence reabsorption and inner-filter effects. Fluorescence emission spectra were recorded at 77 K using LS50B luminescence spectrometer (PerkinElmer, Waltham, MA, USA) equipped with liquid nitrogen sample holder. Chlorophyll fluorescence was excited at 437 nm, and emission spectra were recorded between 660 and 780 nm at 0.5 nm intervals [70,71].

The emission spectra were normalized to the PSII fluorescence maximum (FII) to facilitate comparison among the treatments. The relative contribution of PSI and PSII fluorescence was estimated from the FI/FII ratio [39,72]. In addition, the wavelengths corresponding to the PSI- and PSII-associated fluorescence emission maxima were determined from the normalized spectra to evaluate the senescence-induced changes in photosystem organization and remodeling of chlorophyll–protein complexes.

### 4.6. Gas Exchange Measurements and A/C_i_ Response Curve Analysis

The net CO_2_ assimilation (*A*) responses to the intercellular CO_2_ concentration (*A*/*C*_i_ curves) were measured using an LI-6400XT Portable Photosynthesis System (LI-COR Biosciences, Lincoln, NE, USA) equipped with a 2 × 3 cm chamber and a red–blue LED light source (6400-02B). The measurements were performed at a constant photosynthetic photon flux density (PPFD) of 1500 μmol m^−2^ s^−1^ following stabilization of gas exchange. The leaf temperature was maintained at 23 °C, relative humidity at 60%, flow rate at 500 ± 2 μmol s^−1^, and an ambient atmospheric pressure. The CO_2_ concentration was sequentially adjusted to 400, 300, 200, 100, 50, 400, 400, 600, 800, 1000, 1200, 1400, 1600, 1800, and 2000 μmol mol^−1^ according to the protocol in [73]. The gas exchange parameters were recorded after stabilization at each CO_2_ concentration (120–200 s). The actual leaf area enclosed within the chamber was determined from high-resolution leaf scans using AxioVision 4.8 software (Carl Zeiss, Oberkochen, Germany).

The measured *A*/*C*_i_ response curves were fitted using the Ethier and Livingston model [28] implemented in the *A*/*C*_i_ Curve Fitting tool developed within the LandFlux platform. The fitted model was used to estimate the maximum rate of Rubisco carboxylation (*V*_cmax_), the maximum electron transport rate supporting RuBP regeneration (*J*_max_), the chloroplastic CO_2_ compensation point (*Γ*), and the transition point between Rubisco-limited (*A*_c_) and RuBP regeneration-limited (*A*_j_) photosynthesis. The fitted non-rectangular hyperbola (NRH), together with the *A*_c_ and *A*_j_ limitation curves, were used to identify the predominant biochemical limitation of photosynthesis under each treatment.

### 4.7. Leaf Protein Extraction and Densitometric Quantification

Soluble leaf proteins were extracted from frozen leaf tissue using Plant Total Protein Extraction Kit (Sigma-Aldrich, St. Louis, MO, USA) as described previously [8]. Protein concentration was determined spectrophotometrically using NanoDrop 2000 spectrophotometer (Thermo Fisher Scientific, Waltham, MA, USA). Protein extracts (5 μg protein per lane) were separated on 4–20% TGX gradient polyacrylamide gels (Bio-Rad, Hercules, CA, USA) and stained with Bio-Safe™ Coomassie Brilliant Blue (CBB, Bio-Rad). Three independent biological replicates were analyzed for each treatment. Gel images and band intensities were quantified by densitometric analysis using ImageJ (version 1.53t, National Institutes of Health, Bethesda, MD, USA).

### 4.8. Immunoblot Analysis of Photosynthetic and Senescence-Associated Proteins

Immunoblot analysis was performed essentially as described previously [8]. Following SDS-PAGE, proteins were transferred onto 0.2- or 0.45 μm pore size nitrocellulose membranes (Bio-Rad) by semi-dry electroblotting. Membranes were blocked with 5% (*w*/*v*) non-fat dry milk and incubated with primary antibodies against PsbA (AS05 084, 1:1000, 5 μg protein per lane), Lhcb5 (AS01 009, 1:1000, 5 μg protein per lane), Lhcb1 (AS01 004, 1:1000, 15 μg protein per lane), AtpA (AS08 304, 1:1000, 5 μg protein per lane), cytochrome *f* (PetA) (AS08 306, 1:1000, 15 μg protein per lane), PsaB (AS10 695, 1:1000, 5 μg protein per lane), Lhca4 (AS01 008, 1:1000, 15 μg protein per lane), FNR (AS15 2909, 1:1000, 5 μg protein per lane), PGRL1 (AS10 725, 1:1000, 25 μg protein per lane), NdhS (AS16 4066, 1:1000, 25 μg protein per lane), SAG12 (AS14 2771, 1:1000, 25 μg protein per lane) and eEF1α (AS10 934, 1:5000, 5 μg protein per lane). For PGRL1 immunodetection, protein samples were prepared under non-reducing conditions (without 2-mercaptoethanol; samples were incubated at 70 °C for 10 min prior to electrophoresis) to preserve redox-dependent electrophoretic forms of protein, allowing for reduced (PGRL1_red_) and oxidized (PGRL1_ox_) forms to be resolved as two immunoreactive bands [42]. All primary antibodies were obtained from Agrisera (Vännäs, Sweden). For each target protein, specified amount of total soluble protein was kept constant across all experimental groups within given immunoblot.

Membranes were subsequently incubated with horseradish peroxidase-conjugated goat anti-rabbit IgG secondary antibody (AS09 602, 1:5000–1:10,000; Agrisera). Immunoreactive bands were visualized using Clarity™ Western ECL Substrate (Bio-Rad) and documented with ChemiDoc™ MP Imaging System (Bio-Rad). Band intensities were quantified by densitometric analysis using ImageJ as described above and normalized to corresponding eEF1α loading control prior to statistical analysis [8]. Reduced (PGRL1_red_) and oxidized (PGRL1_ox_) forms of PGRL1, as well as fast- (cyt*f*_f_) and slow-migrating (cyt*f*_s_) forms of cyt*f*, were quantified separately. Three independent biological replicates were analyzed for each treatment.

### 4.9. Statistical Analysis

Data are presented as means ± standard deviation (SD). For each cultivar, differences among treatments were evaluated using one-way analysis of variance (ANOVA), followed by Tukey’s honestly significant difference (HSD) post hoc test at *p* < 0.05. Prior to ANOVA, data were tested for normality using Shapiro–Wilk test and for homogeneity of variances using Levene’s test. Pearson’s correlation coefficients were calculated to evaluate relationships among physiological and biochemical parameters. Correlation heatmaps were generated using OriginPro 2026b (OriginLab Corporation, Northampton, MA, USA). Statistical analyses were performed using STATISTICA 13.3 (TIBCO Software Inc., Palo Alto, CA, USA).

## 5. Conclusions

Dark-induced senescence triggered coordinated functional and structural remodeling of the photosynthetic apparatus in barley, encompassing photosystem activity, photosystem organization, PSI-dependent electron transport, photosynthetic protein composition, and carbon assimilation. Although both cultivars exhibited a comparable decline in PSI photochemistry, they displayed distinct patterns of photosynthetic acclimation. Carina maintained a higher cyclic electron flow despite stronger PSII inhibition, whereas Lomerit exhibited a more pronounced decline in CEF accompanied by an increased PSI acceptor-side limitation. These contrasting responses were associated with the selective remodeling of the PSI–cytochrome *b*_6_*f*–cyclic electron transport module, including coordinated changes in PGRL1, NdhS and cytochrome *f*. Exogenous benzyladenine effectively delayed senescence-associated deterioration of the photosynthetic apparatus by preserving photosystem organization, maintaining PSI-associated proteins and stabilizing regulatory components of cyclic electron transport. The greater responsiveness of Carina to BA coincided with the more effective preservation of PGRL1, cyt*f* and CEF, indicating that the effectiveness of cytokinin-mediated protection largely depends on preservation of the functional integrity of the PSI-associated electron transport network.

Despite the effective preservation of PSI-dependent electron transport, recovery of photosynthetic carbon assimilation remained incomplete, particularly in Carina, where sustained CEF coincided with a pronounced decline in *V*_cmax_. These findings demonstrate that maintenance of cyclic electron transport alone is insufficient to preserve photosynthetic performance unless accompanied by coordinated stabilization of Calvin-cycle metabolism. Collectively, the present study identifies selective remodeling of the PSI-associated electron transport network as a defining feature of dark-induced senescence in barley and its preservation as a key determinant of cytokinin-mediated delay of leaf senescence. The molecular basis underlying the contrasting responses of barley cultivars to cytokinin remains an important subject for future investigation, particularly with respect to cultivar-specific cytokinin perception and signaling.

## Figures and Tables

**Table 1:** Parameters of the non-rectangular hyperbola (NRH) model estimated by fitting the empirical gas exchange *A*/*C*_i_ response data obtained from the first true leaf of *H. vulgare* L. cultivars Carina (C) and Lomerit (L): after 96 h incubation under light preceded by two applications of dH_2_O containing 0.2% DMSO (control, C); after 96 h incubation in darkness preceded by two applications of 50 µM BA in 0.2% DMSO (BA); or after 96 h incubation in darkness preceded by two applications of dH_2_O containing 0.2% DMSO (DIS). The data represent the means ± SD (*n* = 10). Different letters indicate significant differences among the treatments according to Tukey’s HSD test (*p* < 0.05).

	Cultivar and Treatment					
Parameter	C_C	C_BA	C_DIS	L_C	L_BA	L_DIS
*V*_cmax_ * [µmol(CO_2_) m^−2^ s^−1^]	63.41 ± 6.53 ^bc^	13.83 ± 1.46 ^d^	10.57 ± 1.17 ^e^	66.07 ± 7.68 ^ab^	75.17 ± 4.60 ^a^	54.59 ± 7.53 ^c^
*J*_max_ [µmol(electrons) m^−2^ s^−1^]	132.42 ± 12.28 ^b^	35.77 ± 3.32 ^d^	23.43 ± 2.47 ^e^	143.75 ± 12.51 ^ab^	153.08 ± 11.47 ^a^	86.95 ± 8.79 ^c^
*J*_max_/*V*_cmax_	2.09 ± 0.22 ^b^	2.59 ± 0.29 ^a^	2.22 ± 0.14 ^ab^	2.18 ± 0.24 ^ab^	2.04 ±0.16 ^b^	1.59 ± 0.16 ^c^
*A*_c_*= A*_j_ [*C*_i_] [µmol(CO_2_) mol^−1^]	271.93 ± 11.79 ^d^	949.92 ± 38.45 ^a^	859.80 ± 37.29 ^b^	307.17 ± 12.32 ^c^	331.03 ± 14.34 ^c^	81.12 ± 3.52 ^e^
*Γ* [µmol(CO_2_) mol^−1^]	55.55 ± 4.59 ^d^	179.02 ± 16.26 ^b^	330.22 ± 28.15 ^a^	56.01 ± 5.63 ^d^	51.42 ± 6.19 ^d^	81.12 ± 9.02 ^c^

* Abbreviations: *V*_cmax_—maximum Rubisco carboxylation rate; *J*_max_—maximum electron transport rate supporting ribulose-1,5-bisphosphate (RuBP) regeneration; *J*_max_/*V*_cmax_—ratio of maximum electron transport rate to maximum Rubisco carboxylation rate; *A*_c_ = *A*_j_ [*C*_i_]—intercellular CO_2_ concentration at transition point where Rubisco-limited (*A*_c_) and RuBP regeneration-limited (*A*_j_) photosynthesis are equal; *Γ*—chloroplastic CO_2_ compensation point in absence of mitochondrial respiration; *C*_i_—intercellular CO_2_ concentration; RuBP—ribulose-1,5-bisphosphate.

**Table 2:** Relative protein content (AU) estimated by densitometric analysis of bands visualized on gel/membrane following electrophoretic separation of proteins isolated from leaf powder of 50 mm fragments of first true leaf of *H. vulgare* L. cultivars Carina (C) and Lomerit (L) after 72 h incubation under light in 0.2% DMSO (control, C) or in darkness in either 0.2% DMSO (DIS) or in 50 µM BA solution in 0.2% DMSO (BA).

Protein	Relative Abundance [AU] ± SD					
	C_C	C_DIS	C_BA	L_C	L_DIS	L_BA
Rubisco LSU	18.18 ± 0.94 ^c^	18.16 ± 0.43 ^c^	20.12 ± 0.63 ^ab^	20.96 ± 0.74 ^a^	19.20 ± 0.61 ^bc^	20.02 ± 0.27 ^ab^
Rubisco SSU	19.23 ± 0.28 ^c^	19.43 ± 0.08 ^c^	21.76 ± 0.06 ^b^	22.23 ± 0.05 ^a^	21.75 ± 0.14 ^b^	22.34 ± 0.14 ^a^
RCA_total_	41.65 ± 0.57 ^b^	36.31 ± 0.47 ^d^	42.63 ± 0.40 ^b^	46.63 ± 0.34 ^a^	37.38 ± 0.62 ^d^	39.85 ± 0.28 ^c^
SAG12	5.83 ± 0.10 ^c^	21.70 ± 0.82 ^b^	0.00 ± 0.00 ^d^	0.00 ± 0.00 ^d^	23.76 ± 0.63 ^a^	6.63 ± 0.09 ^c^
PsbA	22.24 ± 0.10 ^bc^	22.35 ± 0.07 ^b^	26.32 ± 0.13 ^a^	21.68 ± 0.19 ^cd^	21.94 ± 0.33 ^bd^	21.37 ± 0.33 ^d^
Lhcb5	11.73 ± 0.49 ^c^	6.75 ± 0.24 ^e^	9.30 ± 0.41 ^d^	19.37 ± 0.81 ^a^	13.92 ± 0.33 ^b^	11.92 ± 0.44 ^c^
Lhcb1	13.47 ± 0.13 ^cd^	21.28 ± 0.23 ^a^	15.97 ± 0.17 ^b^	12.29 ± 0.28 ^d^	14.86 ± 1.89 ^bc^	13.89 ± 0.23 ^bd^
AtpA	20.30 ± 0.21 ^a^	16.60 ± 0.21 ^c^	18.20 ± 0.23 ^b^	19.83 ± 0.43 ^a^	16.33 ± 0.28 ^c^	12.62 ± 0.21 ^d^
cyt*f*_s_	1.59 ± 0.06 ^e^	15.12 ± 0.25 ^b^	2.55 ± 0.28 ^cd^	2.16 ± 0.20 ^de^	21.76 ± 0.09 ^a^	2.97 ± 0.42 ^c^
cyt*f*_f_	17.56 ± 0.26 ^c^	19.82 ± 0.23 ^a^	15.10 ± 0.09 ^d^	13.44 ± 0.21 ^e^	15.40 ± 0.40 ^d^	18.55 ± 0.10 ^b^
PsaB	22.45 ± 0.20 ^ab^	21.82 ± 0.29 ^bc^	23.41 ± 0.17 ^a^	21.39 ± 0.45 ^c^	22.53 ± 0.51 ^ab^	21.81 ± 0.43 ^bc^
Lhca4	16.37 ± 0.56 ^b^	14.53 ± 0.38 ^c^	16.00 ± 0.46 ^b^	18.19 ± 0.39 ^a^	13.11 ± 0.28 ^d^	11.90 ± 0.30 ^e^
FNR	18.33 ± 0.34 ^c^	21.71 ± 0.34 ^a^	20.44 ± 0.38 ^b^	20.15 ± 0.48 ^b^	21.77 ± 0.38 ^a^	22.02 ± 0.39 ^a^
PGRL1_red_	12.29 ± 0.39 ^c^	6.54 ± 0.10 ^e^	14.08 ± 0.37 ^b^	15.26 ± 0.35 ^a^	9.32 ± 0.31 ^d^	9.96 ± 0.05 ^d^
PGRL1_ox_	11.55 ± 0.11 ^b^	5.11 ± 0.08 ^e^	10.07 ± 0.17 ^c^	14.11 ± 0.47 ^a^	5.65 ± 0.20 ^e^	6.46 ± 0.30 ^d^
NdhS	9.24 ± 0.33 ^c^	14.76 ± 0.17 ^b^	7.12 ± 0.12 ^d^	9.20 ± 0.38 ^c^	17.27 ± 0.06 ^a^	6.61 ± 0.14 ^d^

The presented values are means of three replicates ± SD. Different superscript letters (a–e) in the same row indicate statistically significant differences among the treatments according to a one-way ANOVA followed by Tukey’s HSD test (*p* < 0.05). The cell colors indicate statistically significant changes relative to the control of the corresponding cultivar. Shades of red denote significant increases, whereas shades of blue denote significant decreases. Color intensity is proportional to the magnitude of the relative change compared to the respective control.

**Table 3:** Equations, definitions and references for photosynthetic electron transport parameters derived from Dual-PAM measurements.

Parameter	Equation	Description	Reference
ΦPSI	ΦPSI = (Pm’ − P)/Pm *	Effective quantum yield of PSI photochemistry	[64]
ΦPSII	ΦPSII = (Fm′ − F)/Fm′	Effective quantum yield of PSII photochemistry in the light-adapted state	[65]
ΦND	ΦND = (P − Po)/Pm	Quantum yield of non-photochemical energy dissipation in PSI due to donor-side limitation (reflecting limitation in electron donation to PSI)	[64]
ΦNA	ΦNA = (Pm − Pm’)/Pm	Quantum yield of non-photochemical energy dissipation in PSI due to acceptor-side limitation (reflecting limitation in electron acceptor availability downstream of PSI)	[64]
NPQ	NPQ = (Fm − Fm′)/Fm′	Non-photochemical quenching of chlorophyll fluorescence in PSII	[66]
ETRI	ETRI = ΦPSI × PAR × Abs × 0.5	Electron transport rate through PSI	[63,67]
ETRII	ETRII = ΦPSII × PAR × Abs × 0.5	Electron transport rate through PSII	[68]
CEF	CEF = ETRI − ETRII	Cyclic electron flow around PSI	[63]
ΦCEF	ΦCEF = ΦPSI − ΦPSII	Effective quantum yield of cyclic electron flow around PSI	[69]

* Abbreviations: P—steady-state P700 signal; Po—minimal P700 signal under far-red light; Pm—maximal oxidizable P700 signal; Pm′—maximal oxidizable P700 signal under actinic light; F—steady-state chlorophyll fluorescence; Fm—maximal chlorophyll fluorescence in dark-adapted state; Fm′—maximal chlorophyll fluorescence in light-adapted state; PAR—photosynthetically active radiation; Abs—leaf absorptance (fraction of incident light absorbed by leaf).

## Data Availability

The data presented in this study are available on request from the corresponding author. The data are not publicly available due to the strict management of various data and technical resources within the research teams.
